# Synergistic Effects of Glutaric Acid With Sorbic Acid and Sodium Bisulphite on Preserving the Quality of Refrigerated Sliced and Minced Salmon

**DOI:** 10.1002/fsn3.70242

**Published:** 2025-05-12

**Authors:** Zhengrui Liao, Xiaotong Zhu, Thaigarajan Parumasivam, Thuan‐Chew Tan, Mohammad Alrosan, Muhammad H. Alu’ datt, Ali Madi Almajwal

**Affiliations:** ^1^ Food Technology Division School of Industrial Technology, Universiti Sains Malaysia Penang Malaysia; ^2^ College of Animal Sciences, South China Agricultural University Guangdong Provincial Key Laboratory of Animal Nutrition Control Guangzhou China; ^3^ School of Pharmaceutical Sciences, Universiti Sains Malaysia Penang Malaysia; ^4^ Renewable Biomass Transformation Cluster School of Industrial Technology, Universiti Sains Malaysia Penang Malaysia; ^5^ QU Health, College of Health Sciences Qatar University Doha Qatar; ^6^ Department of Food Science and Nutrition Faculty of Agriculture, Jerash University Jerash Jordan; ^7^ Department of Food Science and Nutrition College of Life Sciences, Kuwait University Safat Kuwait; ^8^ Department of Community Health Sciences College of Applied Medical Sciences, King Saud University Riyadh Saudi Arabia

**Keywords:** glutaric acid, refrigerated salmon, shelf life, sodium bisulphite, sorbic acid, synergism

## Abstract

The synergistic antimicrobial and antioxidant effects of glutaric acid (GLA) with sorbic acid (SoA) or sodium bisulphite (SoB) were evaluated to determine their optimal concentrations and ratios for maximum efficacy. The results revealed that these combinations exhibited strong antimicrobial properties, with GLA‐SoB also demonstrating significant antioxidant activity. These findings were consistent across both in vitro and food model (using salmon) evaluations. The combinations effectively extended shelf life and preserved freshness when applied to sliced and minced salmon. GLA, GLA‐SoA (2:1), and GLA‐SoB (1:2) were particularly effective in maintaining color, stabilizing pH and moisture levels, and reducing spoilage markers such as total volatile base nitrogen (TVB‐N), peroxide value (POV), thiobarbituric acid (TBA) levels, and microbial counts. This study highlights the comparable effectiveness of GLA‐SoB (1:2) and GLA‐SoA (2:1) in inhibiting microbial growth and extending the shelf life of salmon under refrigerated conditions. Both combinations showed dose‐dependent efficacy in preserving quality and safety, making them promising candidates for enhancing the storage stability of salmon products.

## Graphical abstract

This study evaluates the effectiveness of glutaric acid (GLA) in combination with sorbic acid (SoA) or sodium bisulphite (SoB) for preserving minced and sliced salmon under refrigerated storage. The results indicate that these dual combinations significantly maintain freshness by reducing spoilage indicators such as microbial growth, total volatile base nitrogen (TVB‐N), and weight loss. The findings suggest that GLA‐SoA and GLA‐SoB combinations offer a promising approach to extending salmon shelf life, enhancing both quality and safety in food preservation.

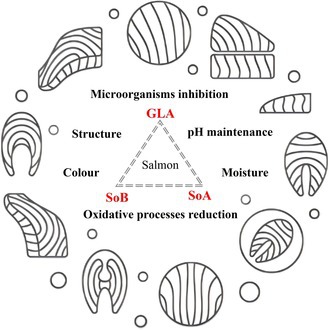

## 1 Introduction

Salmon, renowned for their high commercial value, are widely appreciated for their exceptional quality and nutritional benefits, making them a popular choice in diverse culinary applications (Chen et al. 2023). Due to their perishable nature and limited shelf life, salmon are frequently frozen and maintained in frozen condition during distribution, particularly for long‐distance transportation such as exporting to other countries. To cater to consumers seeking fresh salmon, retailers typically thaw the frozen fish and offer it in various forms, such as salmon slices or minced salmon. Thawed salmon is typically stored at temperatures between 0°C and 4°C to preserve its quality and ensure its safety for consumption until it reaches consumers. However, spoilage can occur during refrigerated storage due to enzymatic activity, microbial growth, and autolytic degradation (Yan et al. 2022). Consequently, chemical preservation techniques are essential for extending shelf life and maintaining the quality of refrigerated salmon. These methods assist in retaining essential attributes, such as texture, color, flavor, nutritional value, and microbiological safety (Yan et al. 2022). While widely employed, chemical preservatives must adhere to local regulatory standards to safeguard consumers from potential adverse effects (Nagasinduja and Shahitha 2019).

In Malaysia, the Food Regulations (1985) authorize the utilization of certain permitted preservatives, including sulfur dioxide (or sulfites) and sorbic acid (or its sodium, calcium, or potassium salts), in uncooked processed meat products, excluding meat burgers (Mavani et al. 2024). These conventional preservatives have been extensively utilized by food industries for an extended period and remain prevalent in contemporary practices under close surveillance of the local authorities. Despite their efficacy, these additives may pose health risks, especially to susceptible individuals, such as asthmatics, even within permissible limits (Lamas et al. 2016).

Organic acids (OAs) are gaining prominence as cost‐effective alternatives for meat preservation, either as standalone agents or in combination with other additives. Their application generally does not necessitate additional processing steps, making them a practical choice for the food industry (Ben Braïek and Smaoui 2021). OAs are widely utilized as natural antimicrobials to inhibit the growth of harmful pathogenic microorganisms such as *Escherichia coli* O157: H7 and *Listeria monocytogenes* . Their salts are also incorporated into ready‐to‐eat products to control pathogens and are applied through washing, spraying, or dipping to sanitize the surfaces of fresh foods (İrkin et al. 2015). While OAs have historically been employed for these purposes, their efficacy and long‐term sustainability necessitate further evaluation and optimization. Notably, combining two or more antimicrobial substances can enhance preservation through additive or synergistic effects, thereby improving the safety and quality of meat products (Ben Braïek and Smaoui 2021). Among the diverse OAs, glutaric acid (GLA) has emerged as a promising compound due to its flavor‐enhancing and stabilizing properties (Hartwig et al. 2020). However, the efficacy of GLA in combination with sorbic acid (SoA) or sodium bisulphite (SoB) in mitigating spoilage and oxidative damage in meat remains uncertain.

The present study investigated the synergistic effects of GLA, SoA, SoB, and combinations of GLA with SoA or SoB on their antimicrobial and antioxidant properties. Through an in vitro approach, the most effective combinations were identified. Subsequently, their efficacy on microbial stability and preservation quality was evaluated using two model systems: refrigerated sliced and minced salmon. In contrast to the sole utilization of conventional preservatives (SoA and SoB) in meat preservation, incorporating GLA can diminish the reliance on these conventional preservatives, enhance food safety, and establish a scientific foundation for further exploration in a broader spectrum of meat products.

## 2 Materials and Methods

### 2.1 Materials

The six microbial strains used to evaluate antimicrobial activity were *Bacillus cereus* (EMCC 1006), *Staphylococcus aureus* (ATCC 25923), *Streptoccus pyogenes* (EMCC 1772), *Candida albicans* (EMCC 105), *Salmonella enterica* (ATCC 43972), and *Escherichia coli* (ATCC 25922). All chemical reagents (including GLA, SoA, and SoB) and solvents used in the study were of analytical grade sourced from Sigma Aldrich (USA).

### 2.2 Determination of Minimal Inhibitory Concentration (MIC)

The MIC was determined following the protocol described by Ben Akacha et al. (2022). The microdilution broth method employed 96‐well microplates to assess the MIC values for each tested substance (GLA, SoA, and SoB). For each well, a 100 μL cell suspension was mixed with a test solution containing the tested substances. The MIC was defined as the lowest concentration of the substance that inhibited visible microbial growth. To confirm the results, 25 μL of thiazolyl blue tetrazolium bromide (MTT) (0.5 mg/mL) was added after 30 min of incubation. Wells with inhibited growth remained clear, indicating no metabolic activity of the tested microbes.

### 2.3 Antimicrobial Efficacy by Checkerboard Assay

The synergistic effects of the tested combinations (GLA combined with either SoA or SoB) were evaluated using the standard checkerboard microdilution method, as described by Xin et al. (2021). Test strains were cultured in Mueller‐Hinton (MH) broth at 37°C until reaching the exponential growth phase. Subsequently, they were diluted to a final concentration of 2 × 10^6^ CFU/mL in fresh MH broth. An 11 × 7 matrix was prepared in a 96‐well microtitre plate by serially diluting one component of the combination (component A) along the rows (50 μL in MH broth) and the other component (component B) along the columns (50 μL in MH broth). A 50 μL microbial inoculum was added to each well, and the plate was incubated at 37°C for 18 h. Following incubation, 50 μL of freshly prepared iodonitrotetrazolium chloride (INT) solution was added to each well, and the plate was further incubated for 2 h at 37°C. MIC values for each combination were determined. The fractional inhibitory concentration (FIC) was calculated as follows:

FIC_A_ = MIC of combination A/MIC of A alone.

FIC_B_ = MIC of combination B/MIC of B alone.

The combined FIC index (∑FIC) was computed as follows:

$$\sum \mathrm{FIC}={\mathrm{FIC}}_{A}+{\mathrm{FIC}}_{B}$$

Interpretation of ∑FIC values was as follows:

∑FIC ≤ 0.5: Synergy, with stronger synergy as values approach zero.

0.5 < ∑FIC ≤ 1: Additivity.

1 < ∑FIC ≤ 2: Indifference.

This method enabled the quantitative evaluation of the interactions between the tested combinations.

### 2.4 Antioxidant Activity

#### 2.4.1 DPPH (2,2‐Diphenyl‐1‐Picrylhydrazyl) Radical‐Scavenging Assay

The DPPH radical‐scavenging activity was determined according to the method described by Hlima et al. (2021). As per Section 3.2, the potency of synergistic interactions between GLA with SoA or SoB is quantified using the ∑FIC index, with values closer to 0 indicating stronger synergy. Consequently, for each strain, the most effective combinations were selected based on a ∑FIC value less than 0.5, demonstrating a robust synergistic effect.

Various ratios of GLA‐SoB (1:1, 1:2, 2:1, 4:1) and GLA‐SoA (2:1, 1:2, 1:4, 1:16) combinations were prepared based on the weight proportions of components A and B. Each tested combination, totalling 4000 μg, was dissolved in 1 mL of 99.5% ethanol, with ascorbic acid (AsA) serving as the positive control. For testing, 500 μL of the combination solution (or 500 μL of distilled water as the negative control) was added to each well of a microplate and mixed with 125 μL of 0.02% DPPH in 99.5% ethanol. AsA was tested in the same manner. An additional 375 μL of 99.5% ethanol was added to reach a final volume of 1 mL, resulting in a solution concentration of 2000 μg/mL. Similarly, solutions at 500 μg/mL and 16,000 μg/mL were prepared for further analysis. The reaction mixture was incubated in the dark for 60 min at room temperature. Following incubation, absorbance was measured at 517 nm with ethanol as the blank using a UV–visible spectrophotometer (PG Instruments, T70, Shanghai, China). A control sample was prepared by mixing the DPPH solution with ethanol. The DPPH radical‐scavenging activity was calculated using the following formula:

$\text{DPPH radical}-\text{scavenging activity}\;\left(\%\right)=\left[1-\left(A_{S}/A_{C}\right)\right]\times 100\%$

Where, *A*_C_ represents the absorbance of the negative control (distilled water), and *A*_S_ is the absorbance of the sample.

#### 2.4.2 Nitrite Scavenging Activity In Vitro

The nitrite‐scavenging activity assay was conducted according to the protocol outlined by Choi et al. (2019). A total of 1 mL of each sample solution (containing 9 mg of studied substances in 1 mL 99.5% ethanol) or 1 mL of 99.5% ethanol (serving as a blank) was mixed with 1 mL of citric acid buffer (pH 3) and 1 mL of a 5 mg/L nitrate solution. The resulting mixture was incubated at 37°C for 30 min. Subsequently, 1 mL of a 4 g/L sodium amino benzene sulfonic acid solution (in 20% hydrochloric acid) and 0.5 mL of a 2 g/L naphthalene ethylenediamine hydrochloride solution (in water) were added to reach a final concentration of 2000 μg/mL. Solutions with concentrations of 500 and 16,000 μg/mL were prepared similarly. The reaction was rested for 15 min before measuring the absorbance at 538 nm. AsA was utilized as the positive control, and the experiment was performed in triplicate. The nitrite‐scavenging capacity was calculated using the following equation:

$\text{Nitrite}-\text{scavenging capacity}\;\left(\%\right)=\left(1-A_{S}/A_{B}\right)\times 100\%$

Where, *A*_S_ is the absorbance of the sample and *A*_B_ is the absorbance of the blank.

### 2.5 Tested Combination Treatments Using Salmon as Model Systems

#### 2.5.1 Sliced Salmon Model System

The preparation of sliced salmon samples followed the method outlined by Yan et al. (2022). Fresh salmon ( *Salmo salar* ), sourced from a Norwegian fish farm (Bremnes Seashore AS, Bømlo), was purchased from a local market (Lotus's, Pulau Pinang, Malaysia). The salmon were subsequently sliced to uniform dimensions of approximately 41.0 ± 4.3 mm in length, 19.0 ± 2.9 mm in width, and 6.0 ± 0.3 mm in thickness. The samples were divided into 17 groups for treatment: Groups 1–3 (treated with SoB), Groups 4–6 (treated with SoA), Groups 7–9 (treated with GLA), Groups 10–12 (treated with GLA‐SoB 1:2), and Groups 13–15 (treated with GLA‐SoA 2:1), with each group receiving treatments at concentrations of 500, 1000, and 2000 μg/mL, respectively. Group 16 was treated with 40% ethanol as a blank control, while Group 17 was left untreated as a negative control. Each slice was subsequently immersed in the respective treatment solution (dissolved in 40% ethanol) for 1 min, followed by air drying at 35°C for 15 min using a drying oven (Thermo Scientific, Heratherm, Waltham, USA). The treated and control samples were subsequently stored at 4°C in a refrigerated incubator (Thermo Scientific, FYCD‐290, Waltham, USA), and quality and safety analyses were performed on days 1, 3, 6, 9, and 12.

#### 2.5.2 Minced Salmon Model Study

The average weight of each sliced salmon sample was determined to be approximately 3 g, as per the procedures outlined in Section 2.5.1. The weight (*M*) of each tested combination (in μg) applied per 30 g of minced salmon was calculated using the following equations:

$$\text{∆}W=W_{t}-W_{0}$$

V=∆W/ρ

m=V×c

M=10×m

where, *W*_t_ and *W*_0_ represent the weight (in g) of the treated and untreated slices, respectively, ∆*W* represents the weight of the solution adhering to the sliced salmon surface (in g), *ρ* represents the relative density of 40% ethanol (0.92 g/mL), *V* represents the solution volume (in mL), *c* represents the solution concentration (in μg/mL), and *m* represents the weight of the tested combination adhering to a single slice (in μg).

Minced salmon samples were prepared according to the method outlined by Pakawatchai et al. (2009). Fresh minced salmon was obtained from a Lotus's market and divided into 22 test groups: Groups 1–4 (SoB), Groups 5–8 (SoA), Groups 9–12 (GLA), Groups 13–16 (GLA‐SoB 1:2), and Groups 17–20 (GLA‐SoA 2:1), with each group treated at concentrations of 182, 364, 728, and 1456 μg/g. Group 21 served as a blank control with 40% ethanol, and Group 22 was an untreated negative control. Each test group was treated with 5 mL of the corresponding solution with 40% ethanol as the solvent. Samples were stored at 4°C in the FYCD‐290 refrigerated incubator, and quality and safety evaluations of the samples were conducted on days 1, 3, 6, 9, and 12.

### 2.6 Quality and Safety Assessments of Salmon Samples

#### 2.6.1 Microbial Analysis

Microbial analysis was conducted following Abdel‐Wahab et al. (2020). A 25 g salmon sample was homogenized at room temperature for 90 s using a stomacher (Autoscience, ATBM‐400B, Tianjin, China). A 10 g portion of the homogenate was mixed with 90 mL of sterile peptone solution (25.5 g/L), and 100 μL of this mixture was plated on various media for microbial enumeration using serial dilutions from 10^−1^ to 10^−7^. Total viable counts (TVC) were determined on Plate Count Agar incubated at 37°C for 48 h. Psychrotrophic bacterial counts (PTC) were determined on Plate Count Agar incubated at 7°C for 10 days. Yeast and mold counts were determined on Potato Dextrose Agar incubated at 25°C for 96 h. Enterobacteriaceae counts were determined on Violet Red Bile Glucose Agar incubated at 37°C for 48 h.

#### 2.6.2 Colourimetric Properties

The color characteristics of the salmon samples, including lightness (L*), redness (a*), and yellowness (b*), were evaluated using a colorimeter (Konica Minolta, CR‐200, Tokyo, Japan).

#### 2.6.3 pH Measurement

The pH levels of the salmon samples were determined using a calibrated pH meter (Lutron, YK‐21PH, Taiwan, China). For sample preparation, 3 g of homogenized salmon was blended with 30 mL of distilled water. The pH of the resulting filtrate was measured according to the procedure outlined by Hsouna et al. (2022).

#### 2.6.4 Determination of Weight Loss

The initial (*W*_i_) and final weights (*W*_f_) of the salmon samples were recorded before and after the storage period. The weight loss percentage was calculated using the following equation:

$\text{Weight loss}\;\left(\%\right)=\left[\left(W_{i}-W_{f}\right)/W_{f}\right]\times 100\%.$

#### 2.6.5 Total Volatile Base Nitrogen (TVB‐N) Determination

TVB‐N levels in the salmon samples were analyzed following the procedure described by Jia et al. (2021). The samples were homogenized in a stomacher (Seward, 400 sq., West Sussex, British) with distilled water at a 1:10 (w/v) ratio. A 5 mL aliquot of the supernatant was mixed with 5 mL of 10 g/L MgO solution and distilled using a Kjeldahl nitrogen analyzer (Alva Instrument, KN‐520, Jinan, China). The distillate was collected in a boric acid solution (20 mL, 0.02 g/L) containing a mixed indicator (methyl red and methylene blue, 1 g/L each in ethanol). The solution was then titrated with 0.01 M HCl. A blank sample using 5 mL of distilled water in place of the salmon sample was also tested. The TVB‐N content was calculated using the following equation:

$\mathrm{TVB}-N\;\left(\mathrm{mg}/100\;g\right)=\left[\left(V_{1}–V_{2}\right)\times 0.01\times 14/\left(m\times 5/50\right)\right]\times 100$

Where, *m* represents the sample weight (in g), *V*_1_ and *V*_2_ represent the volumes (in mL) of HCl used for the sample and blank, respectively, 0.01 represents the concentration of the HCl solution (in mol/L), and 50 represents the total sample volume (in mL) in the mixture.

#### 2.6.6 Determination of Thiobarbituric Acid (TBA) Value

The TBA value of the salmon samples was determined according to the method outlined by Dirpan and Hidayat (2023). A 3 g salmon sample was blended with 50 mL of distilled water and homogenized in the 400 sq. stomacher for 2 min. The homogenate was transferred into a 1000 mL distillation flask and rinsed with an additional 48.5 mL of distilled water. HCl (1.5 mL) was added, and the mixture was heated for 10 min, resulting in 50 mL of distillate. The distillate was filtered and mixed with 5 mL of TBA reagent (0.02 M thiobarbituric acid in 90% glacial acetic acid). This solution was heated in a boiling water bath (PWB‐4, Boeco, Stuttgart, Germany) for 35 min to enhance the reaction, followed by cooling in cold water. The absorbance of the solution was measured at 528 nm using the T70 spectrophotometer, with a blank sample serving as the baseline.

#### 2.6.7 Determination of Primary Oxidation Products Using Peroxide Value (POV)

Salmon samples were extracted to obtain a supernatant prior to the determination of POV based on the procedure performed previously by Manihuruk et al. (2017). Initially, salmon samples (50 g) were homogenized in the 400 sq. stomacher with absolute methanol at a 1:5 ratio (w/v) for 20 s at room temperature. The homogenate was filtered using filter paper to obtain the supernatant, which was then stored in sealed bottles at‐20°C until further analysis.

For lipid separation, a biphasic solvent system comprising water, methanol, and chloroform in a 25:100:100 ratio was used, following the Wu et al. (2016) lipid extraction method. The stored supernatant was mixed with this solvent system and vigorously shaken to promote phase separation. After standing, two distinct layers formed: the upper aqueous phase containing water‐soluble compounds and the lower organic phase rich in lipids dissolved in chloroform. The lipid‐containing organic phase was carefully collected and transferred to a separate container for concentration.

The extracted lipids were concentrated using a rotary evaporator (Yarong Biochemical Analysis Instrument, RE‐5220, Shanghai, China) to remove solvents under reduced pressure. A 1 g portion of the concentrated lipids was then dissolved in 6 mL of an acetic acid/chloroform solution (3:2). To initiate the reaction, 0.1 mL of saturated potassium iodide was added, and the mixture was left to react in the dark for 10 min. Subsequently, 10 mL of distilled water and 0.1 mL of a 1% starch solution were introduced. The mixture was then titrated with 0.01 M sodium thiosulfate (Na_2_S_2_O_3_) until it became colorless. The POV was calculated and expressed in milliequivalents (meq) per kilogram of lipid, as per the following equation:

$\mathrm{POV}=\left(N\times V/W\right)\times 100$

Where, *N* represents the normality of Na_2_S_2_O_3_, *V* represents the volume of Na_2_S_2_O_3_, and *W* represents the weight of the lipid extract.

### 2.7 Statistical Analysis

Statistical analyses were conducted using GraphPad Prism (version 6.01). A one‐way analysis of variance (ANOVA) followed by Tukey's post hoc test was employed for multiple comparisons. All experiments were performed in triplicate (*n* = 3), and results are presented as mean ± standard deviation (SD). Statistical significance was deemed at *p* < 0.05.

## 3 Results and Discussion

### 3.1 Antimicrobial Activities of Different Tested Combinations

Table 1 presents the MIC values of the tested substances against specific microorganisms. Among them, SoA exhibited the highest MIC (2000 μg/mL) for most microorganisms, except *Streptococcus pyogenes* . This was followed by GLA, which exhibited an MIC of 1000 μg/mL against *Candida albicans* . Lastly, SoB demonstrated an MIC of 500 μg/mL against *Escherichia coli* , *Bacillus cereus* , and *Candida albicans* . These findings highlight the significance of choosing suitable antimicrobial substances tailored to the target microorganisms, as resistance levels can differ significantly. This underscores the need for precise treatments, optimal concentrations, or strategic combinations to achieve effective inhibition.

**TABLE 1:** Minimum inhibitory concentrations (MIC, μg/mL) of the tested substances, sodium bisulphite (SoB), glutaric acid (GLA), sorbic acid (SoA) against specific microorganisms.

Studied substances	*Salmonella enterica* ATCC 43972	*Escherichia coli* ATCC 25922	*Streptococcus pyogenes* EMCC 1772	*Bacillus cereus* EMCC 1006	*Staphylococcus aureus* ATCC 25923	*Candida albicans* EMCC 105
SoB	250	500	250	500	125	500
GLA	500	250	500	250	250	1000
SoA	2000	2000	1000	2000	2000	2000

*Note:* The test was carried out in triplicate (*n* = 3).

Previously, Seo et al. (2023) identified *Escherichia coli* , *Salmonella enterica* , and *Staphylococcus aureus* as the most resistant bacteria to SoA, with an MIC of 2000 μg/mL. In contrast, earlier research indicates that the MIC values of these microorganisms for SoB range from 300 to 50,000 μg/mL (Brennan et al. 1999; Lamas et al. 2016; Pardeshi et al. 2015; Quoc 2018; Rojo‐Bezares et al. 2007). This potential difference in results may be attributed to resistance mechanisms associated with prior exposure to preservatives or antibiotics (Krishnamoorthy et al. 2021), leading to notable variations compared to the findings in this study. Moreover, to the best of our knowledge, no studies have reported the MIC values of the tested microorganisms against GLA (Table 1), underscoring the need for further research into its inhibitory properties.

The antimicrobial activity of dual combinations (GLA with SoA or SoB) was assessed against various microbial strains (Table 2). In instances where the ∑FIC was less than 0.5, the MIC of each substance was reduced to as low as 1/4 of its single‐agent MIC, or even lower. A ∑FIC index closer to 0 indicates a more substantial synergistic effect. Promising combinations include GLA‐SoB at ratios of 1:1, 1:2, 2:1, and 4:1, as well as GLA‐SoA at ratios of 1:2, 1:4, 1:16, and 2:1.

**TABLE 2:** The antimicrobial effects of glutaric acid (GLA) combined with sodium bisulphite (SoB) or sorbic acid (SoA) against different microbial strains.

Strains	Combination substances			Parameters					
	A	B	A:B^a^	MIC_A_ (μg/mL) in combination	MIC_B_ (μg/mL) in combination	FIC_A_	FIC_B_	∑ FIC	Interaction
Bacteria Gram−									
*Salmonella enterica* ATCC 43972	GLA	SoB	1:2	7.8125	15.625	0.016	0.063	0.079	Synergism
			1:2	15.625	31.25	0.031	0.125	0.156	Synergism
	GLA	SoA	8:1	125	15.625	0.25	0.008	0.258	Synergism
			2:1	62.5	31.25	0.125	0.016	0.141	Synergism
*Escherichia coli* ATCC 25922	GLA	SoB	4:1	62.5	15.625	0.25	0.031	0.281	Synergism
			1:2	31.25	62.5	0.125	0.125	0.25	Synergism
	GLA	SoA	8:1	125	15.625	0.5	0.008	0.508	Additivity
			1:2	62.5	125	0.25	0.063	0.313	Synergism
Bacteria Gram+									
*Streptococcus pyogenes* EMCC 1772	GLA	SoB	1:2	7.8125	15.625	0.016	0.063	0.079	Synergism
	GLA	SoA	4:1	62.5	15.625	0.125	0.008	0.133	Synergism
			1:4	31.25	125	0.063	0.063	0.126	Synergism
*Staphylococcus aureus* ATCC 25923	GLA	SoB	1:1	31.25	31.25	0.125	0.25	0.375	Synergism
			1:1	15.625	15.625	0.063	0.125	0.188	Synergism
	GLA	SoA	8:1	125	15.625	0.5	0.016	0.516	Additivity
			1:2	62.5	125	0.25	0.125	0.375	Synergism
			1:16	31.25	500	0.125	0.5	0.625	Additivity
*Bacillus cereus* EMCC 1006	GLA	SoB	8:1	125	15.625	0.5	0.031	0.531	Additivity
			2:1	62.5	31.25	0.25	0.063	0.313	Synergism
			1:4	31.25	125	0.125	0.25	0.375	Synergism
			1:16	15.625	250	0.063	0.5	0.563	Additivity
	GLA	SoA	8:1	125	15.625	0.5	0.008	0.508	Additivity
			2:1	62.5	31.25	0.25	0.016	0.266	Synergism
			1:2	31.25	62.5	0.125	0.031	0.156	Synergism
			1:16	7.8125	125	0.031	0.063	0.094	Synergism
Yeast									
*Candida albicans* EMCC 105	GLA	SoB	4:1	62.5	15.625	0.063	0.031	0.094	Synergism
	GLA	SoA	2:1	62.5	31.25	0.063	0.063	0.126	Synergism
			1:2	31.25	62.5	0.031	0.125	0.156	Synergism
			1:8	15.625	125	0.016	0.25	0.266	Synergism

*Note:* The test was carried out in triplicate (*n* = 3).

^a^ Weight ratio.

All tested combinations exhibited nearly synergistic or additive effects against the selected microbes. These findings align with those of Kim and Rhee (2013), who reported that four distinct short‐chain fatty acids and three medium‐chain fatty acids, including caprylic acid, exhibited synergistic effects against pathogenic bacteria such as *Escherichia coli* . This was evident from the more significant reduction in microbial populations compared to individual treatments. Currently, no studies have explored the mechanisms underlying the synergism between GLA and SoA or SoB. However, several mechanisms have been proposed, such as (a) sequential inhibition of multiple steps within a biochemical pathway, (b) suppression of protective enzymes, and (c) interactions with the cell wall or membrane that increase the uptake of other antimicrobial agents (Chaichi et al. 2021). The synergistic effects observed in the present study may result from the diverse substances within the applied combinations, each acting on different critical targets in or on the cell wall, thereby enhancing microbial control. The interaction between GLA and SoA may specifically be attributed to an increase in the undissociated form of the acid, which amplifies proton release. This cumulative effect overwhelms microbial buffering and transport systems, leading to more excellent inhibitory action than a single acid (Ben Braïek and Smaoui 2021). Similarly, the various GLA‐SoB combinations may target multiple mechanisms, such as disrupting intracellular pH, impairing protein functions, destabilizing cell membranes, and ultimately inhibiting microbial growth synergistically (Dhakal et al. 2019). GLA combinations can achieve an ∑FIC of less than 0.1 against *Salmonella enterica* , *Streptococcus pyogenes* , and *Candida albicans* , suggesting heightened sensitivity to their accumulation. In contrast, other microbes listed in Table 2 appear more tolerant, possibly due to their ability to incorporate exogenous GLA into metabolic processes, which may reduce its efficacy (Chaichi et al. 2021).

Moreover, combining the tested substances offers the advantage of reducing the required concentrations of each component to achieve effective antimicrobial activity. This approach could provide significant benefits, enhancing efficacy while potentially reducing costs and minimizing the adverse effects of higher doses of SoB and SoA.

### 3.2 Evaluation of Antioxidant Activities of Tested Combinations

The nitrite‐scavenging capacity and DPPH assays were employed to evaluate the total antioxidant activity of the tested combinations (Table 3). Both antioxidant assays produced comparable results. The assays indicated that the GLA‐SoA combination exhibited the most substantial synergistic antioxidant effect at a 2:1 ratio. In contrast, the GLA‐SoB combination exhibited its highest synergistic antioxidant activity at a 1:2 ratio.

**TABLE 3:** Synergistic effects of glutaric acid (GLA), sodium bisulfite (SoB), sorbic acid (SoA), and their combinations on antioxidant activity.

Proportion of combinations	Studied substances	DPPH scavenging activity at 500 μg/mL (%)	DPPH scavenging activity at 2000 μg/mL (%)	DPPH scavenging activity at 16,000 μg/mL (%)	Nitrite scavenging at 500 μg/mL (%)	Nitrite scavenging at 2000 μg/mL (%)	Nitrite scavenging at 16,000 μg/mL (%)
	AsA	82.38 ± 1.97^a^	91.15 ± 2.64^a^	98.71 ± 0.90^a^	96.99 ± 1.73^a^	98.13 ± 0.43^a^	98.28 ± 0.36^a^
	SoB	75.92 ± 1.05^a^	88.52 ± 1.95^ab^	98.76 ± 0.25^a^	95.51 ± 1.21^a^	95.84 ± 1.71^a^	98.20 ± 0.28^a^
	GLA	15.01 ± 3.71^f^	16.34 ± 4.08^de^	28.34 ± 0.64^e^	50.91 ± 1.93^f^	53.91 ± 1.88^d^	55.86 ± 2.88^de^
	SoA	10.51 ± 1.23^i^	11.38 ± 1.45^e^	18.38 ± 1.45^g^	30.62 ± 0.68^i^	31.29 ± 1.35^g^	35.00 ± 1.93^h^
1:1	GLA‐SoB	63.62 ± 1.02^d^	74.62 ± 2.84^bc^	95.28 ± 0.51^b^	90.11 ± 0.80^c^	89.11 ± 1.86^b^	88.09 ± 1.40^b^
2:1	GLA‐SoB	42.29 ± 1.65^e^	70.13 ± 3.42^c^	86.43 ± 1.04^c^	65.86 ± 2.30^d^	71.86 ± 0.74^c^	63.30 ± 1.69^c^
4:1	GLA‐SoB	21.77 ± 1.50^f^	66.10 ± 6.33^c^	76.22 ± 1.79^c^	51.66 ± 1.74^e^	54.66 ± 2.25^d^	58.42 ± 1.06^d^
1:2	GLA‐SoB	69.65 ± 1.33^c^	80.38 ± 4.52^b^	97.71 ± 0.62^a^	94.22 ± 1.31^b^	95.55 ± 1.58^a^	96.16 ± 1.52^a^
2:1	GLA‐SoA	12.84 ± 1.81^g^	18.67 ± 3.92^d^	23.01 ± 3.16^f^	44.23 ± 1.79^g^	51.89 ± 4.07^de^	57.80 ± 1.79^d^
1:2	GLA‐SoA	12.01 ± 2.01^g^	16.32 ± 4.92^de^	18.94 ± 0.43^g^	41.96 ± 1.81^g^	47.29 ± 2.97^ef^	54.07 ± 1.53^e^
1:4	GLA‐SoA	11.65 ± 1.17^g^	16.11 ± 4.46^de^	17.65 ± 4.35^g^	39.57 ± 1.23^h^	45.76 ± 1.73^f^	50.03 ± 0.63^f^
1:16	GLA‐SoA	10.94 ± 2.11^g^	12.27 ± 3.43^de^	16.44 ± 5.31^g^	38.76 ± 1.73^h^	45.23 ± 1.50^f^	41.01 ± 1.25^g^

*Note:* The test was carried out in triplicate (*n* = 3). Significant changes (*p* < 0.05) are indicated by different letters during the same assay.

Both SoB and GLA, when present at a concentration of 500 μg/mL, effectively inhibited most of the tested microorganisms (Table 1). Notably, all three tested substances exhibited inhibitory effects at a concentration of 2000 μg/mL in vitro. SoA and its salts are classified as GRAS (Generally Recognized as Safe) additives. While these preservatives represent a safe option, there are potential safety concerns for specific vulnerable populations (Stopforth and Kudron 2020). As per National Health Commission of the People's Republic of China and State Administration for Market Regulation (2024), the permissible maximum concentration of SoA in meat products is 1500 μg/g. Testing concentrations of 500, 2000, and 16,000 μg/mL (equivalent to 1456 μg/g, which falls within the legal limit) were calculated in Section 2.5.2 and provided valuable insights into the feasible application ranges of these substances in the subsequent salmon meat storage models.

Although SoA is widely utilized for its antimicrobial properties, it lacks the antioxidant capabilities of ascorbic acid (vitamin C) or tocopherols (vitamin E) (Meyer et al. 2002). Antioxidants typically possess functional groups, such as hydroxyl (‐OH) groups, which facilitate electron donation or hydrogen atom transfer to neutralize free radicals and disrupt oxidative chain reactions. In contrast, SoA lacks these reactive groups and is incapable of readily engaging in redox reactions to stabilize free radicals (Meyer et al. 2002). Consequently, SoA is frequently combined with antioxidants in food and beverage preservation when oxidative stability is paramount (Stopforth and Kudron 2020). GLA shares similar limitations. Its structure, characterized by the presence of only two carboxyl (‐COOH) groups, lacks substituents capable of participating in redox reactions with free radicals, rendering it ineffective at preventing oxidative chain reactions (Liao et al. 2024). Therefore, the combination of GLA with SoA does not significantly augment antioxidant capacity. In contrast, SoB exhibits distinct behavior. As a reducing agent, SoB can donate electrons to neutralize oxidizing compounds, effectively halting oxidative chain reactions. When SoB is combined with GLA, the resulting mixture exhibits enhanced antioxidant capacity, with the effect becoming more pronounced as the SoB concentration increases (Vellanki et al. 2013).

### 3.3 Salmon Product Preservation

#### 3.3.1 Evaluation of Quality Parameters in Sliced Salmon Treated With Tested Combinations

**FIGURE 1:**
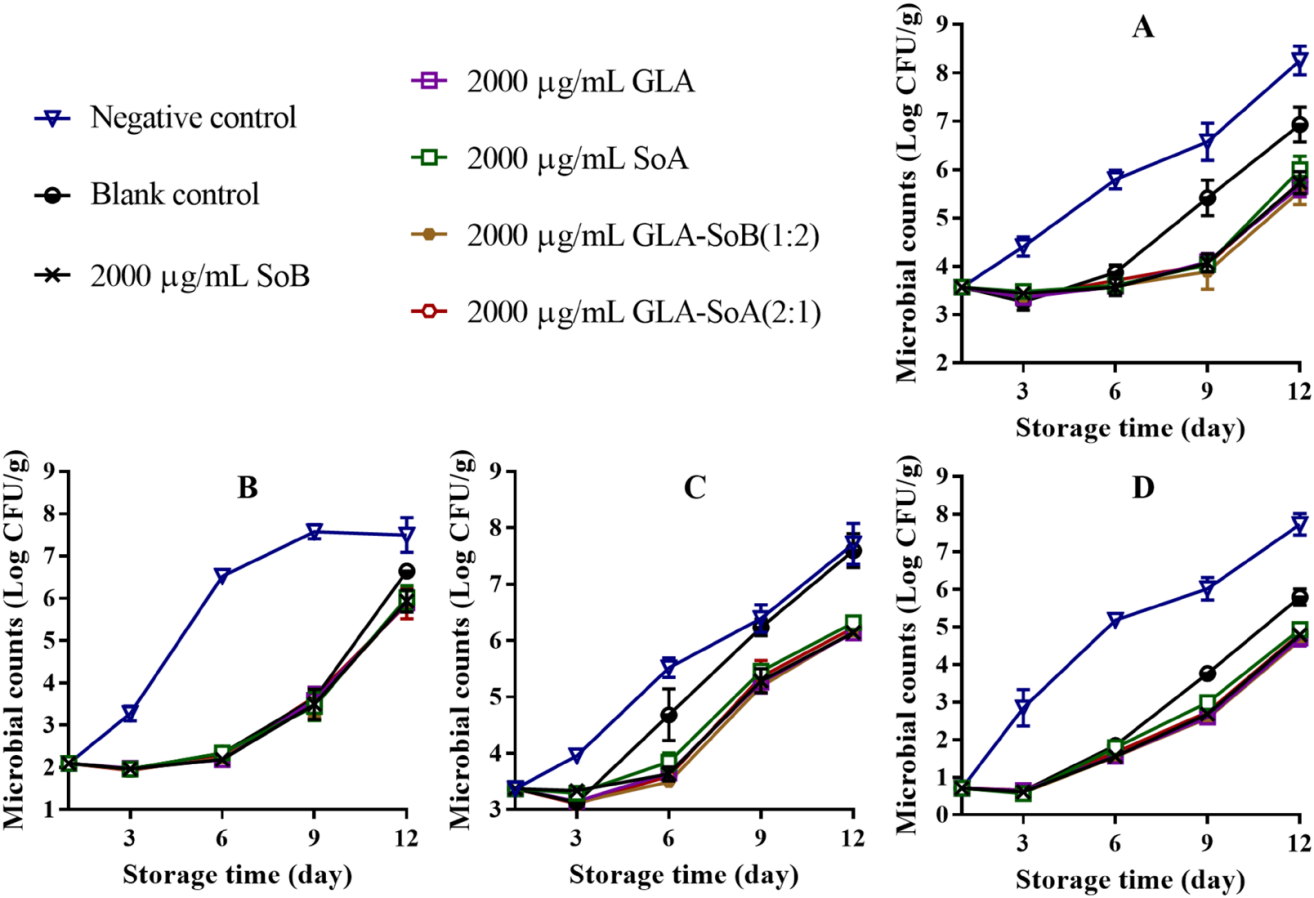
Assessment of total viable count (A), psychrophilic bacteria count (B), yeasts and molds count (C), and Enterobacteriaceae count (D) in sliced salmon treated with glutaric acid (GLA), sodium bisulphite (SoB), sorbic acid (SoA) and their combinations at 2000 μg/mL during storage at 4°C. The blank control group represents sliced salmon treated solely with 40% ethanol. Values represent the mean ± SD of three replicates.

**FIGURE 2:**
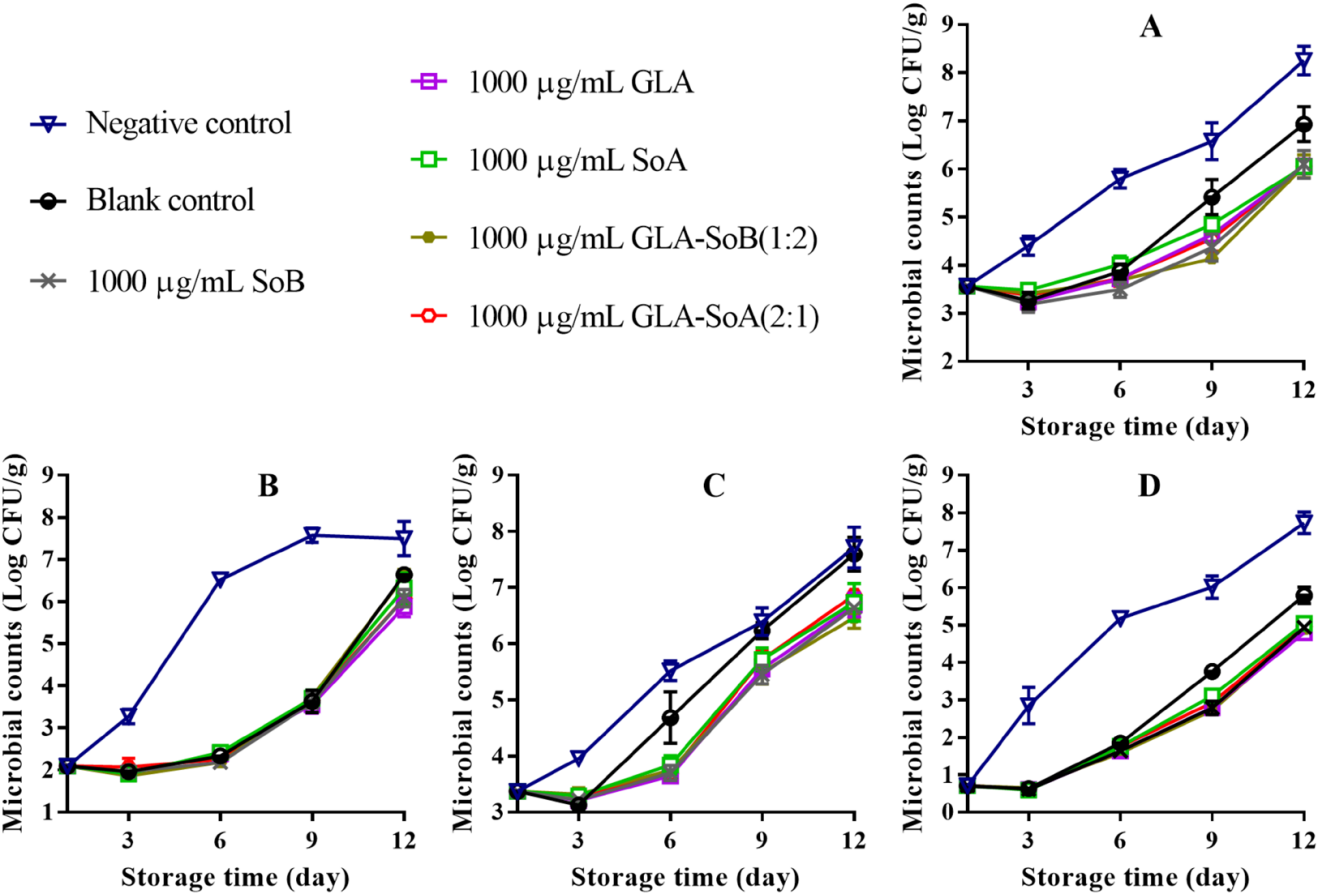
Assessment of total viable count (A), psychrophilic bacteria count (B), yeasts and molds count (C), and Enterobacteriaceae count (D) in sliced salmon treated with glutaric acid (GLA), sodium bisulphite (SoB), sorbic acid (SoA) and their combinations at 1000 μg/mL during storage at 4°C. The blank control group represents sliced salmon treated solely with 40% ethanol. Values represent the mean ± SD of three replicates.

**FIGURE 3:**
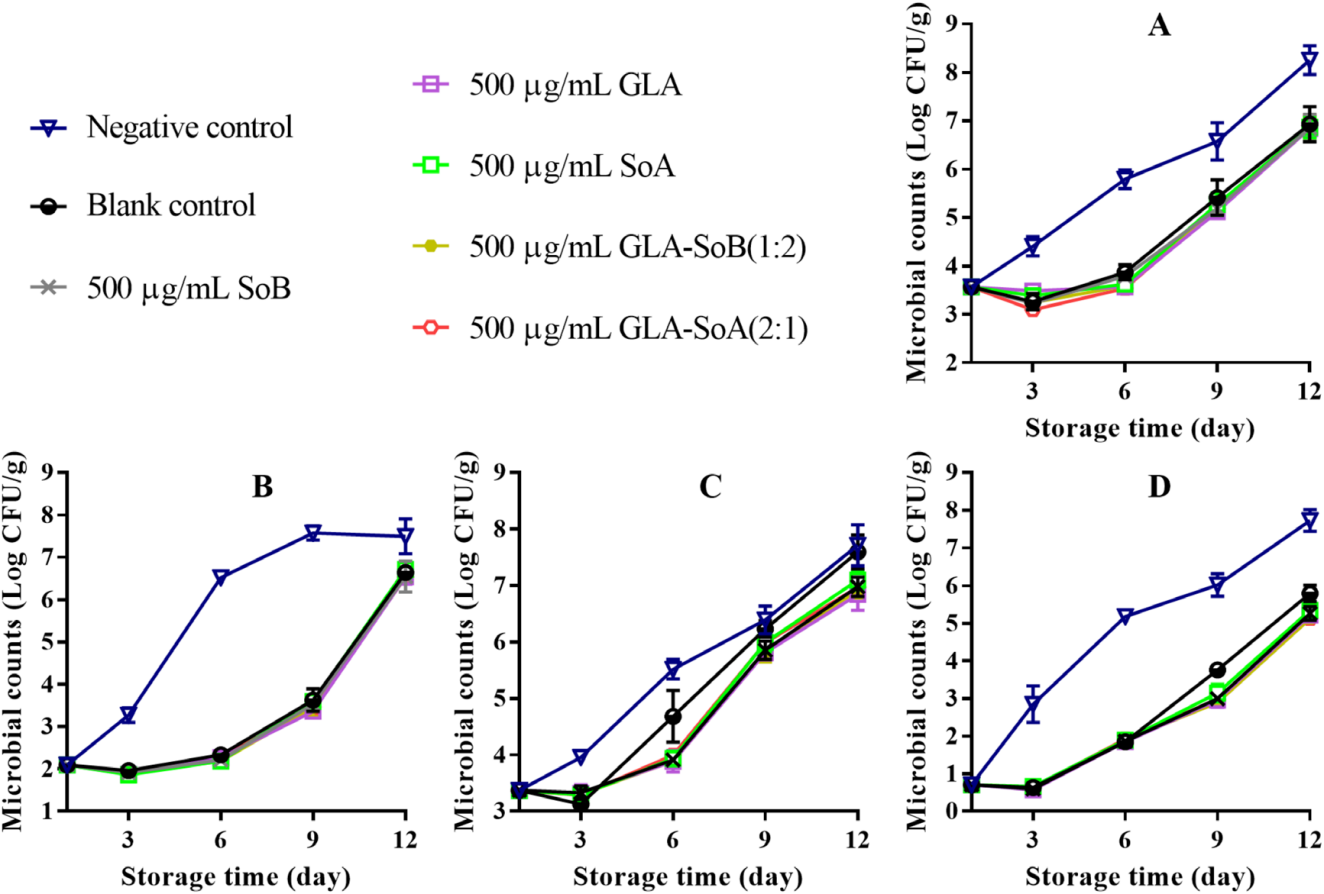
Assessment of total viable count (A), psychrophilic bacteria count (B), yeasts and molds count (C), and Enterobacteriaceae count (D) in sliced salmon treated with glutaric acid (GLA), sodium bisulphite (SoB), sorbic acid (SoA) and their combinations at 500 μg/mL during storage at 4°C. The blank control group represents sliced salmon treated solely with 40% ethanol. Values represent the mean ± SD of three replicates.

Figures 1, 2, 3 illustrate the changes in TVC, PTC, Enterobacteriaceae count, and yeast and mold count during refrigerated storage of sliced salmon. The initial TVC of the sliced salmon ranged from 3.52 to 3.60 log_10_ CFU/g, indicative of the high quality of the salmon used in the present study. Throughout storage, TVCs, PTCs, Enterobacteriaceae counts, and yeast and mold counts exhibited variations across all experimental groups. Notably, microbial counts in the negative and blank control groups exhibited significant increases (*p* < 0.05) compared to those in the experimental groups after day 9. Notably, the TVCs in the negative and blank control groups surpassed the acceptable limit of 7.0 log_10_ CFU/g (Wu et al. 2016) by approximately days 9 and 12, respectively. In contrast, the GLA‐SoB (1:2) and GLA‐SoA (2:1) treatments demonstrated microbial inhibition comparable (*p* > 0.05) to SoB, GLA, and SoA at the end of the storage period. Furthermore, the TVCs in these experimental groups remained below the acceptable limit of 7.0 log_10_ CFU/g for 12 days after storage commenced. These findings suggest that the tested substances, particularly the GLA‐SoB (1:2) and GLA‐SoA (2:1) combinations, extended the microbiological shelf life of sliced salmon by at least 3 days compared to the negative control.

These findings align with previous reports. Karami et al. (2020) observed that minced trout samples coated with chitosan‐flaxseed mucilage films exhibited a microbial population reduction of 0.35–4.91 log_10_ CFU/g compared to control samples after 16 days of refrigeration, consistent with the findings of this research. Similarly, Olivas et al. (2003) demonstrated that immersion in an aqueous solution containing 1% ascorbic acid, 0.25% calcium chloride, and 0.1% potassium sorbate provided bacteriostatic effects and extended the shelf life of treated samples. Furthermore, a treatment involving a 3% SoB solution, either alone or combined with 200 μg/mL of peracetic acid, applied via a 15‐s dip to chicken drumsticks effectively reduced *Salmonella* presence. This reduction was evident after 3 days of refrigeration, suggesting that the combined action of SoB and peracetic acid effectively controlled *Salmonella* growth during storage (Dittoe et al. 2019). These findings support the antimicrobial efficacy of SoB when used alone, indicating that combining SoB with OAs can enhance its impact. The antimicrobial properties of OAs are influenced by chain length, branching degree, and the proportion of undissociated forms. Acidification, partly due to the bisulphite ions (HSO^3−^) released by SoB, contributes to its enhanced antimicrobial activity. This mechanism has been extensively documented in the literature (İrkin et al. 2015). OA combinations play a critical role in preservation by lowering pH, which amplifies antimicrobial effectiveness. A reduction in the number of microorganisms is directly correlated with a decrease in food spoilage, as microbial activity is a primary driver of deterioration (Dittoe et al. 2019). Notably, when SoA and SoB were combined with GLA, the concentrations required for preserving sliced salmon and extending their shelf life were significantly reduced compared to using SoA or SoB alone.

**FIGURE 4:**
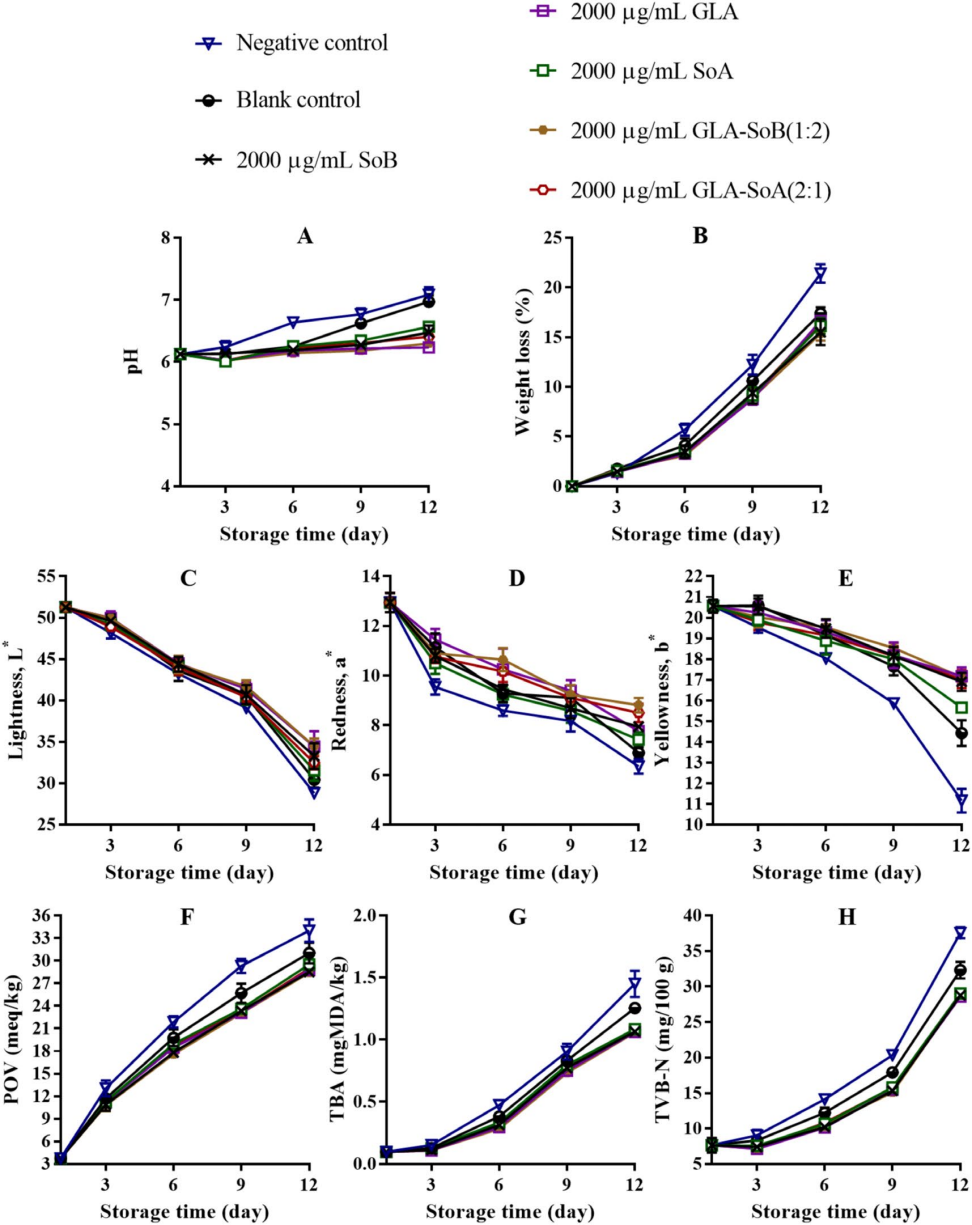
Assessment of pH (A), weight loss (B), lightness (C), redness (D), yellowness (E), peroxide value (POV) (F), thiobarbituric acid (TBA) value (G), and total volatile basic‐nitrogen (TVB‐N) (H) of sliced salmon treated with glutaric acid (GLA), sodium bisulphite (SoB), sorbic acid (SoA) and their combinations at 2000 μg/mL during storage at 4°C. The blank control group represents sliced salmon treated solely with 40% ethanol. Values represent the mean ± SD of three replicates.

**FIGURE 5:**
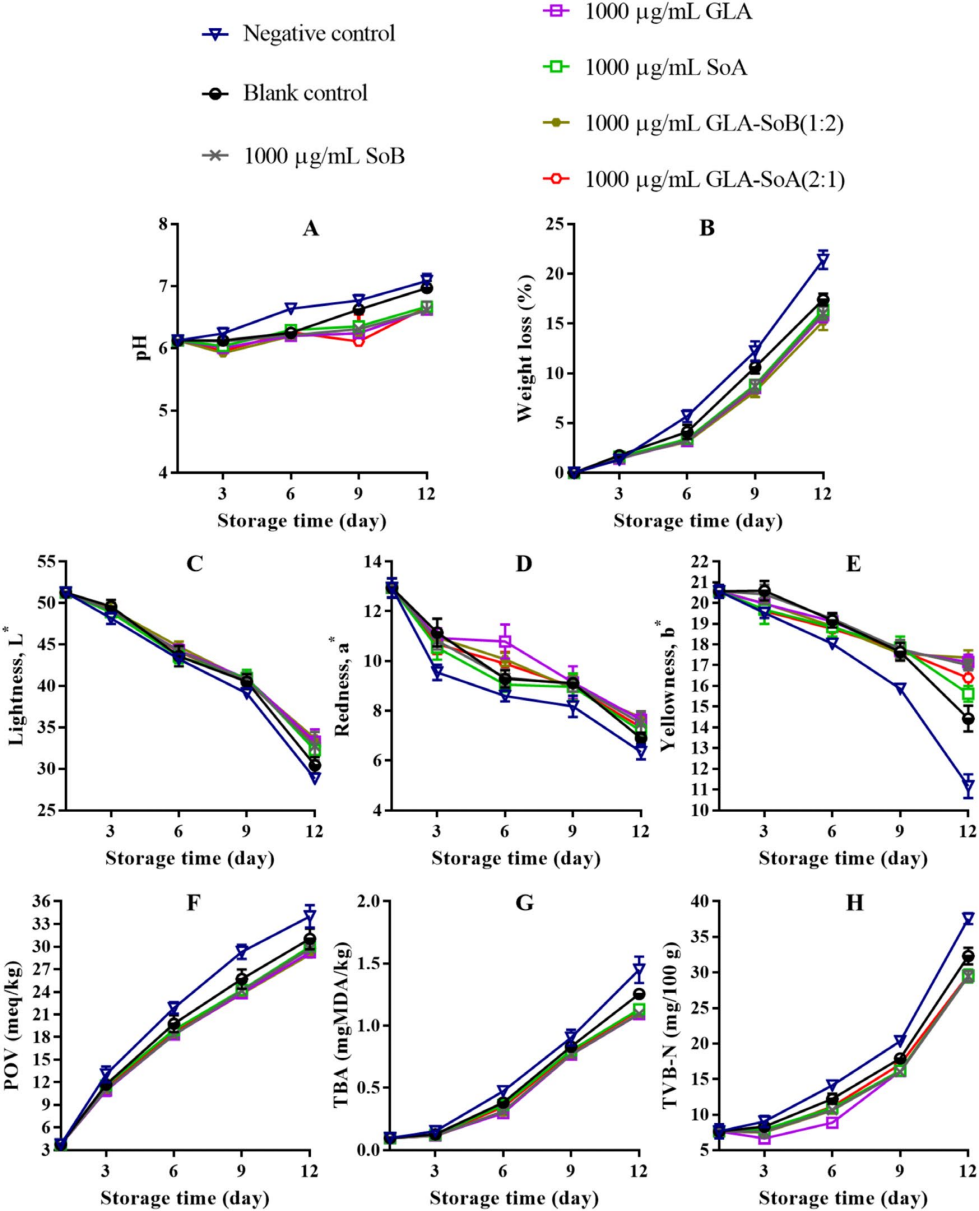
Assessment of pH (A), weight loss (B), lightness (C), redness (D), yellowness (E), peroxide value (POV) (F), thiobarbituric acid (TBA) value (G), and total volatile basic‐nitrogen (TVB‐N) (H) of sliced salmon treated with glutaric acid (GLA), sodium bisulphite (SoB), sorbic acid (SoA) and their combinations at 1000 μg/mL during storage at 4°C. The blank control group represents sliced salmon treated solely with 40% ethanol. Values represent the mean ± SD of three replicates.

**FIGURE 6:**
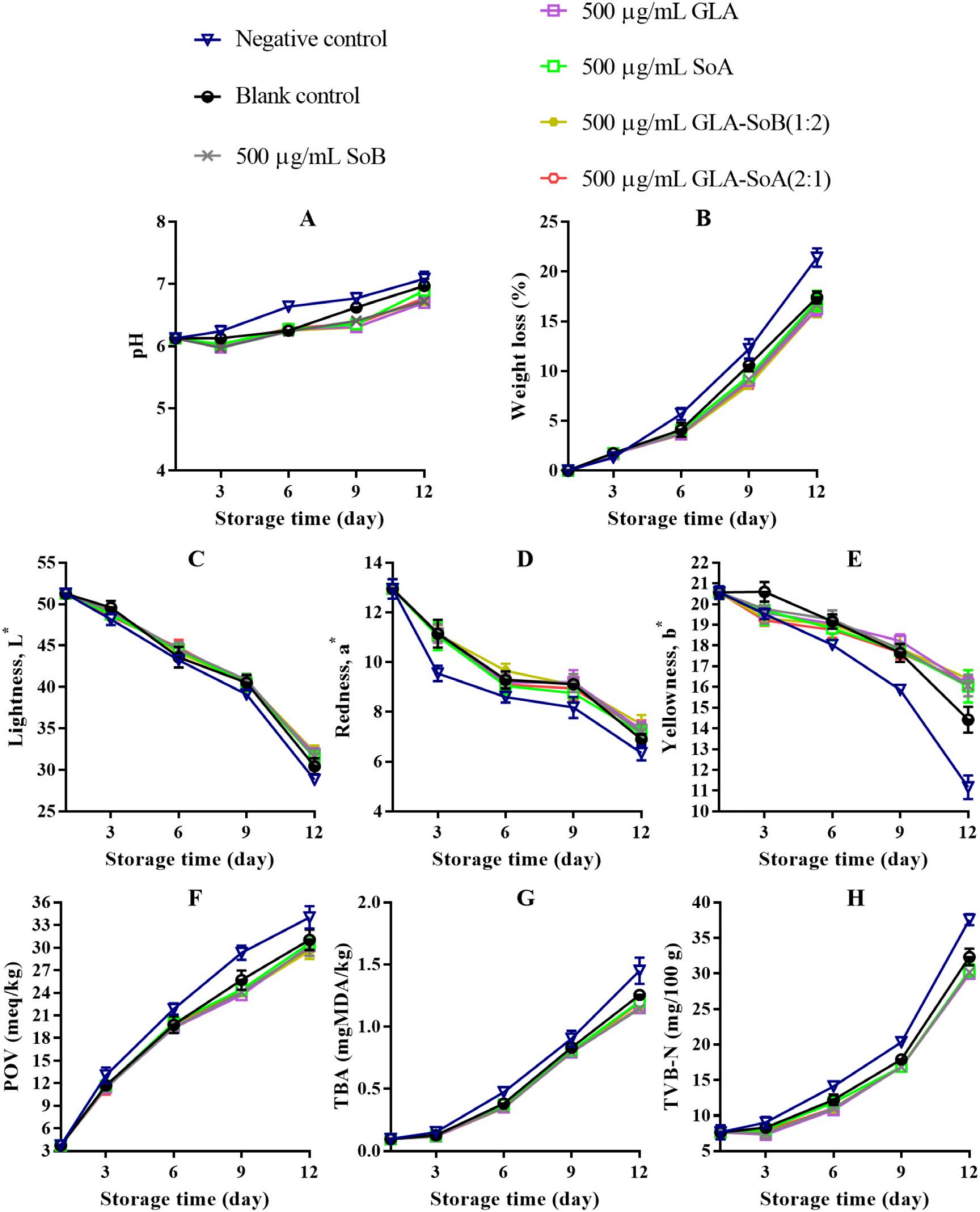
Assessment of pH (A), weight loss (B), lightness (C), redness (D), yellowness (E), peroxide value (POV) (F), thiobarbituric acid (TBA) value (G), and total volatile basic‐nitrogen (TVB‐N) (H) of sliced salmon treated with glutaric acid (GLA), sodium bisulphite (SoB), sorbic acid (SoA) and their combinations at 500 μg/mL during storage at 4°C. The blank control group represents sliced salmon treated solely with 40% ethanol. Values represent the mean ± SD of three replicates.

The pH of sliced salmon exhibited a gradual increase across all treatments during refrigerated storage, with the magnitude of this rise being more pronounced in the negative and blank control groups (Figures 4A, 5A, and 6A). This pH elevation can be attributed to the accumulation of alkaline compounds generated by microbial activity during the post‐rigor phase. Notably, the tested combinations demonstrated the capacity to mitigate post‐mortem deterioration through their antimicrobial effects (Section 3.1). Following previous research conducted by Alves et al. (2018), salmon samples generally tended to rise in pH during refrigerated storage. However, samples treated with GLA combinations exhibited consistently low pH values throughout the storage period, likely due to the release of H^+^ ions from GLA and SoA. A dose‐dependent relationship was observed, with lower concentrations, particularly 500 μg/mL, exhibiting reduced stability in maintaining pH levels. This suggests that the GLA‐SoB (1:2) and GLA‐SoA (2:1) combinations effectively minimized pH increases. A decrease in pH plays a crucial role in preserving the food matrix by establishing a more acidic environment that inhibits microbial growth and enzymatic activity. Lower pH levels effectively retard the proliferation of spoilage organisms and pathogenic bacteria, thereby safeguarding the texture, flavor, and overall quality of the food (Alves et al. 2018). Furthermore, a stable acidic environment can augment the efficacy of certain preservatives and antioxidants, thereby extending shelf life and ensuring food safety during storage (Dittoe et al. 2019).

In all treatments, weight loss decreased progressively throughout the refrigerated storage period (Figures 4B, 5B and 6B). This reduction in weight loss is associated with protein denaturation and the subsequent exposure of hydrophobic groups. Similar trends have been observed by Molina et al. (2014) in cultured sea bass fillets ( *Dicentrarchus labrax* ) and by Christensen et al. (2017) in mackerel loins. Samples treated with SoB, GLA, SoA, GLA‐SoB (1:2), and GLA‐SoA (2:1) exhibited significantly (*p* < 0.05) lower weight loss compared to the negative control group throughout the storage period. No significant differences (*p* > 0.05) in weight loss were detected among samples treated with these five treatments, indicating comparable efficacy in minimizing weight loss during refrigerated storage.

Protein denaturation in refrigerated meat is indirectly linked to microbial spoilage. Microbial activity produces metabolic byproducts, such as proteolytic enzymes, which degrade proteins (Anas et al. 2019). Additionally, protein denaturation during refrigeration can reduce water‐holding capacity, resulting in drip loss. This creates a moist surface that fosters microbial growth and accelerates spoilage (Ismail and Huda 2024). The reduction in weight loss supports the structural integrity of the food matrix, indicating that moisture retention is preserved, which is essential for maintaining texture and quality (Ismail and Huda 2024). This stability also suggests that microbial activity is effectively suppressed, as many spoilage microorganisms break down key nutrients such as proteins and fats, leading to degradation, water loss, and textural changes. By inhibiting microbial growth, the food retains its original composition for a longer period, preventing excessive moisture evaporation and nutrient breakdown, ultimately enhancing its shelf life and freshness (Lerfall et al. 2016).

Proteins in meat are also susceptible to oxidative damage caused by reactive oxygen species (ROS) (Domínguez et al. 2021). SoB, when combined with GLA, neutralizes ROS, mitigating oxidative modification of proteins that could otherwise lead to denaturation. Antioxidants counteract oxidative damage and often exhibit antimicrobial properties, further reducing microbial activity and the associated proteolytic enzyme production (Domínguez et al. 2021). In addition to its antimicrobial effects, SoB may also assist in stabilizing the color of sliced salmon when used in conjunction with GLA, contributing to overall quality preservation during storage.

Color measurements of all samples were conducted on days 1, 3, 6, 9, and 12 (Figures 4C−E, 5C−E, and 6C−E). Lightness (L*) values exhibited a progressive decline during storage, with a more rapid reduction observed in the negative and blank control groups. In contrast, treated samples demonstrated better control of L* reduction after day 1. Redness (a*) values decreased gradually throughout storage, with the rate of decline being most pronounced in the negative control group, followed by the blank control and experimental groups. Significant differences (*p* < 0.05) were detected among all treated samples before and after storage. On day 12, GLA‐SoB (1:2) at 2000 μg/mL retained the highest redness levels, followed by GLA‐SoA (2:1) at 2000 μg/mL, and subsequently by individual treatments of GLA, SoB, and SoA at 2000 μg/mL. Yellowness (b*) values also decreased significantly (*p* < 0.05) during storage. However, treated samples maintained superior retention of b* values compared to untreated controls, corroborating with previous findings in salmon meat (Christensen et al. 2017). These results underscore the efficacy of the tested combinations in preserving the color quality of salmon during refrigerated storage.

Both antioxidant action and microbial inhibition play vital roles in improving and maintaining the color of fish meat during refrigeration. Oxidative degradation of lipids generates byproducts that interact with proteins and pigments, adversely affecting color (Comi 2017). By reducing lipid oxidation, SoB, in combination with GLA, effectively preserves the natural appearance of sliced salmon. Spoilage bacteria contribute to discoloration by producing metabolites, such as hydrogen sulfide, and enzymes that degrade proteins and pigments. Controlling microbial growth prevents these processes, maintaining a fresher and more aesthetically pleasing appearance (Comi 2017). The synergistic effect of SoB and GLA enhances preservation, as their complementary actions address both oxidative and microbial pathways leading to discoloration. In contrast, the combined use of SoA and GLA amplifies their synergistic effect, primarily targeting the microbial pathways responsible for salmon discoloration. These dual‐action approaches demonstrate exceptional efficacy in protecting the color and quality of refrigerated sliced salmon.

Lipid oxidation in seafood products is typically measured by POV and TBA methods. The impact of the treatments on salmon lipid oxidation is depicted in Figures 4F, 5F and 6F. The initial POV of the negative and blank control samples exhibited a significant (*p* < 0.05) increase over the storage period. conversely, the treated samples, including those exposed to 40% ethanol alone, demonstrated stronger resistance to oxidation (*p* < 0.05) compared to the negative control group. TBA analysis, which indicates the content of secondary lipid oxidation products, revealed a gradual increase in TBA values across all groups over time (Figures 4G, 5G and 6G). Treated sliced salmon exhibited significantly (*p* < 0.05) lower TBA values compared to the controls, particularly after day 9 during the 12‐day storage period. This effect was more pronounced at 2000 and 1000 μg/mL treatment concentrations. Consistent with the POV results, samples treated with the tested combinations, namely GLA‐SoB (1:2) and GLA‐SoA (2:1), exhibited low TBA values at the end of storage, indicating effective mitigation of lipid oxidation.

Previous research has established a correlation between POV levels and psychrotrophic bacterial growth. These bacteria produce lipase and phospholipase enzymes during refrigeration, facilitating the oxidation of released short‐chain fatty acids (Li et al. 2021). Furthermore, the observed differences in TBA values between treatments with the tested combinations and the negative control are likely attributed to their bactericidal, antioxidant, or combined effects. This observation aligns with findings by Basavegowda and Baek (2021), who demonstrated that compounds exhibiting bactericidal or antioxidant properties can inhibit the formation and oxidation of unsaturated fats, the primary drivers of rancidity in food products. By reducing oxidation, the tested combinations effectively preserve the quality and stability of sliced salmon during storage, preventing rancidity and extending shelf life.

Figures 4H, 5H and 6H present the TVB‐N values, a marker of bacterial decomposition in fish meat, in relation to the storage period. Initial TVB‐N levels in sliced salmon ranged from 7.05 to 7.71 mg N/100 g. Progression of TVB‐N values was observed in all samples, with significant (*p* < 0.05) differences emerging between the control and treated groups after day 3. The rate of TVB‐N increase was notably slower in samples treated with tested combinations at 2000 and 1000 μg/mL, that is, GLA‐SoB (1:2) and GLA‐SoA (2:1). Before day 12, the TVB‐N value of the negative control exceeded 35 mg/100 g, surpassing the previously established maximum threshold. As reported by Alves et al. (2018), a TVB‐N level of 35 mg/100 g is considered the upper limit for acceptable salmon freshness. In contrast, by day 12, these treated groups exhibited significantly (*p* < 0.05) lower TVB‐N values compared to the controls, attributed to their high antimicrobial efficacy (Section 3.1). A strong correlation was observed between TVB‐N, PTC, and TVC values, further substantiating the efficacy of these treatments in preserving salmon quality during refrigerated storage (Jia et al. 2021). When these results are integrated with the results presented in Figures 1, 2, 3, the tested combinations effectively reduce TVC, PTC, and other microbial counts. This aligns with the observed decreases in TVB‐N, TBA, and POV, suggesting a potential correlation. Furthermore, the reduction in these parameters may also be attributed to the suppression of microbial catabolism (Basavegowda and Baek 2021).

#### 3.3.2 Evaluation of Quality Parameters in Minced Salmon Treated With Tested Combinations

**FIGURE 7:**
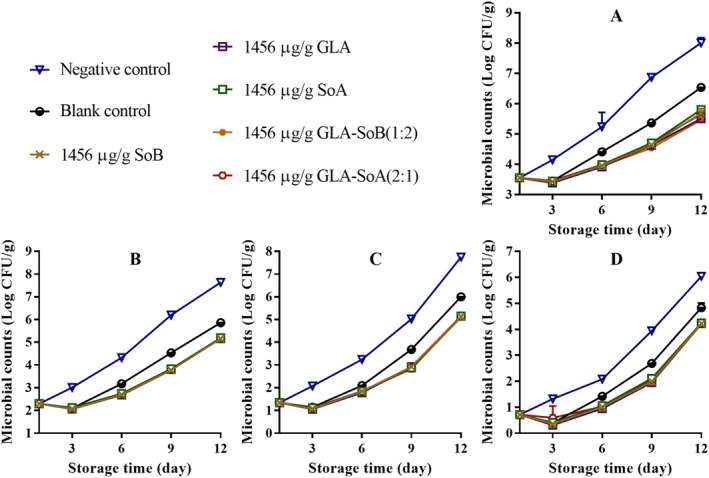
Assessment of total viable count (A), psychrophilic bacteria count (B), yeasts and molds count (C), and Enterobacteriaceae count (D) in minced salmon treated with glutaric acid (GLA), sodium bisulphite (SoB), sorbic acid (SoA) and their combinations at 1456 μg/g during storage at 4°C. The blank control group represents minced salmon treated solely with 40% ethanol. Values represent the mean ± SD of three replicates.

**FIGURE 8:**
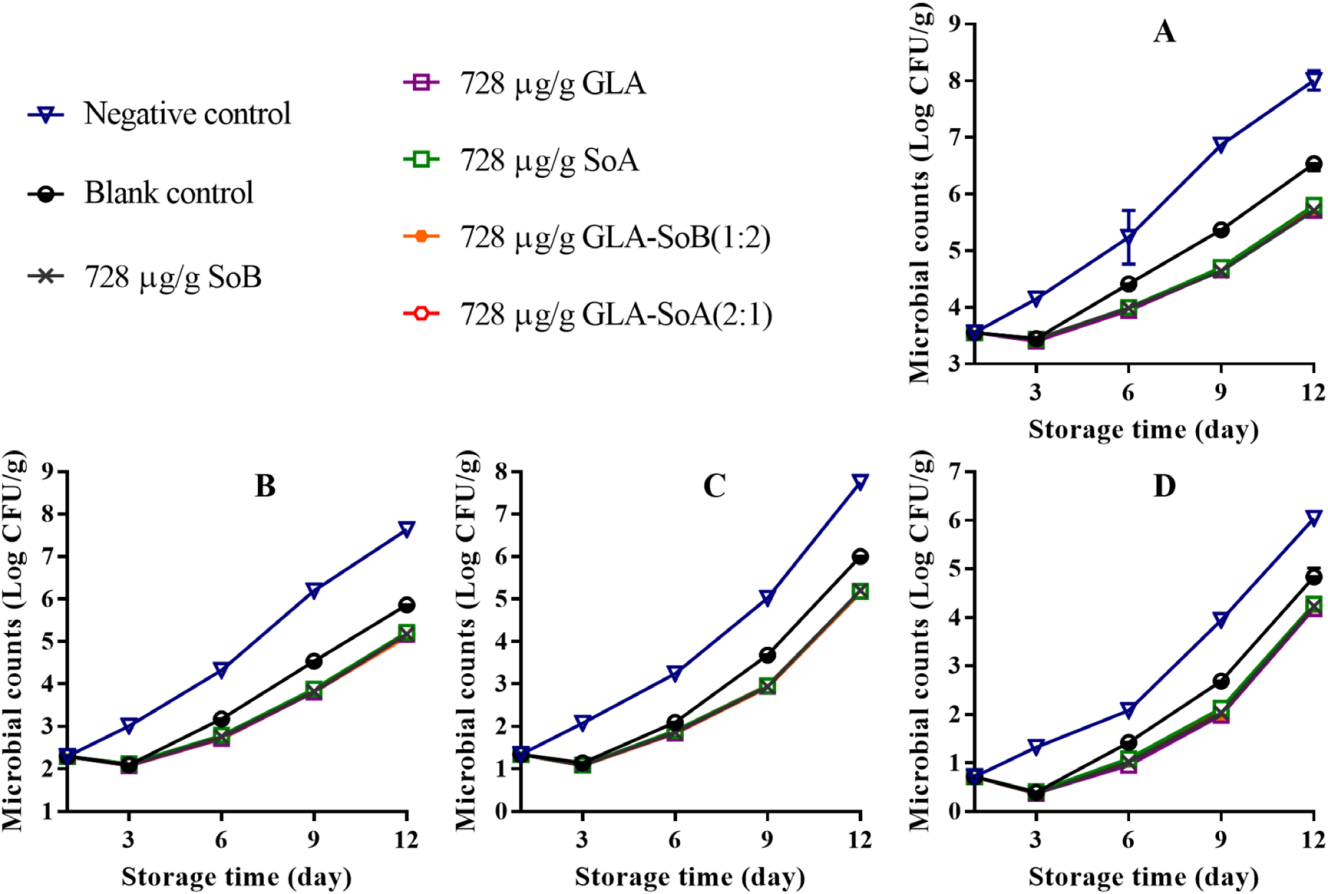
Assessment of total viable count (A), psychrophilic bacteria count (B), yeasts and molds count (C), and Enterobacteriaceae count (D) in minced salmon treated with glutaric acid (GLA), sodium bisulphite (SoB), sorbic acid (SoA) and their combinations at 728 μg/g during storage at 4°C. The blank control group represents minced salmon treated solely with 40% ethanol. Values represent the mean ± SD of three replicates.

**FIGURE 9:**
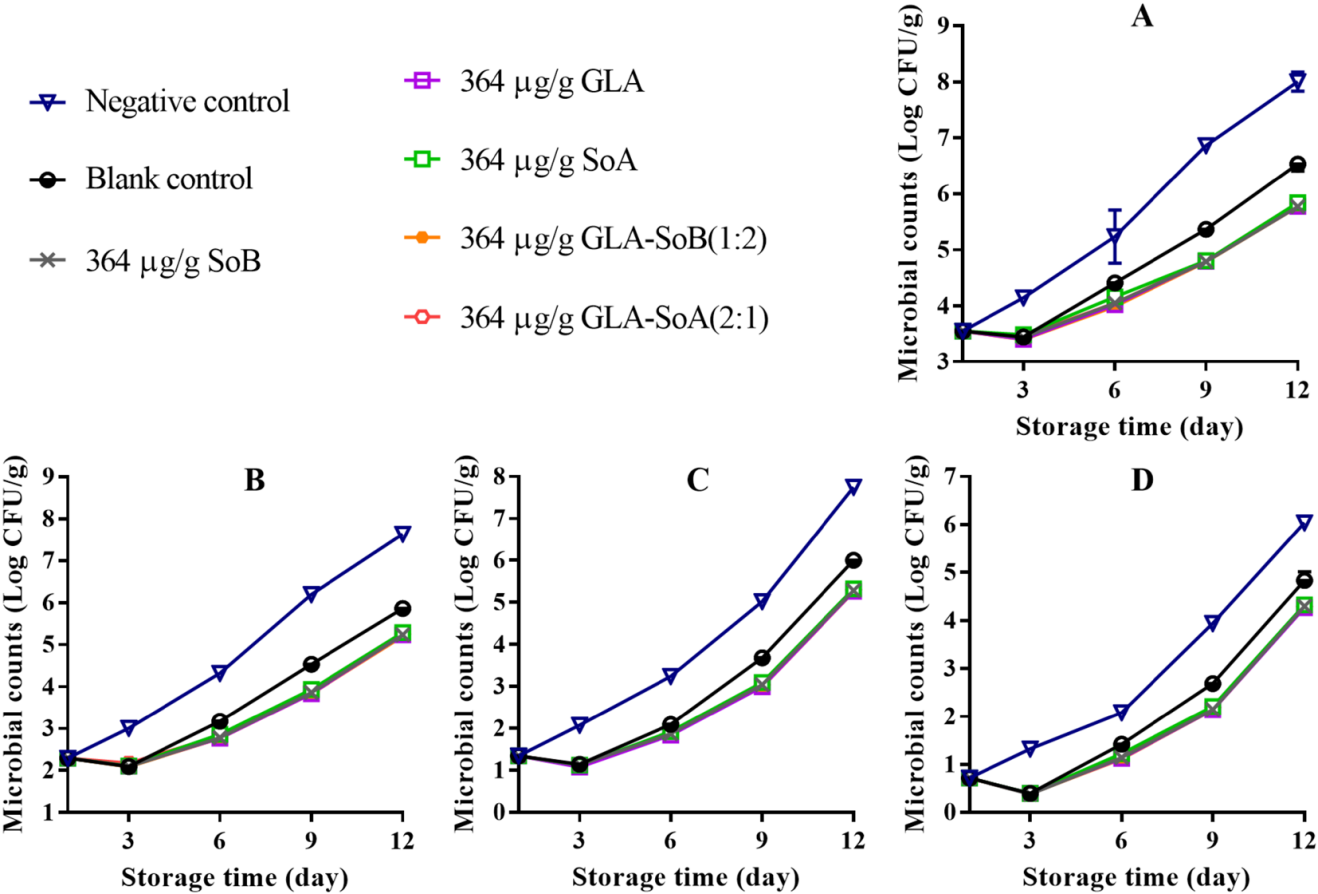
Assessment of total viable count (A), psychrophilic bacteria count (B), yeasts and molds count (C), and Enterobacteriaceae count (D) in minced salmon treated with glutaric acid (GLA), sodium bisulphite (SoB), sorbic acid (SoA) and their combinations at 364 μg/g during storage at 4°C. The blank control group represents minced salmon treated solely with 40% ethanol. Values represent the mean ± SD of three replicates.

**FIGURE 10:**
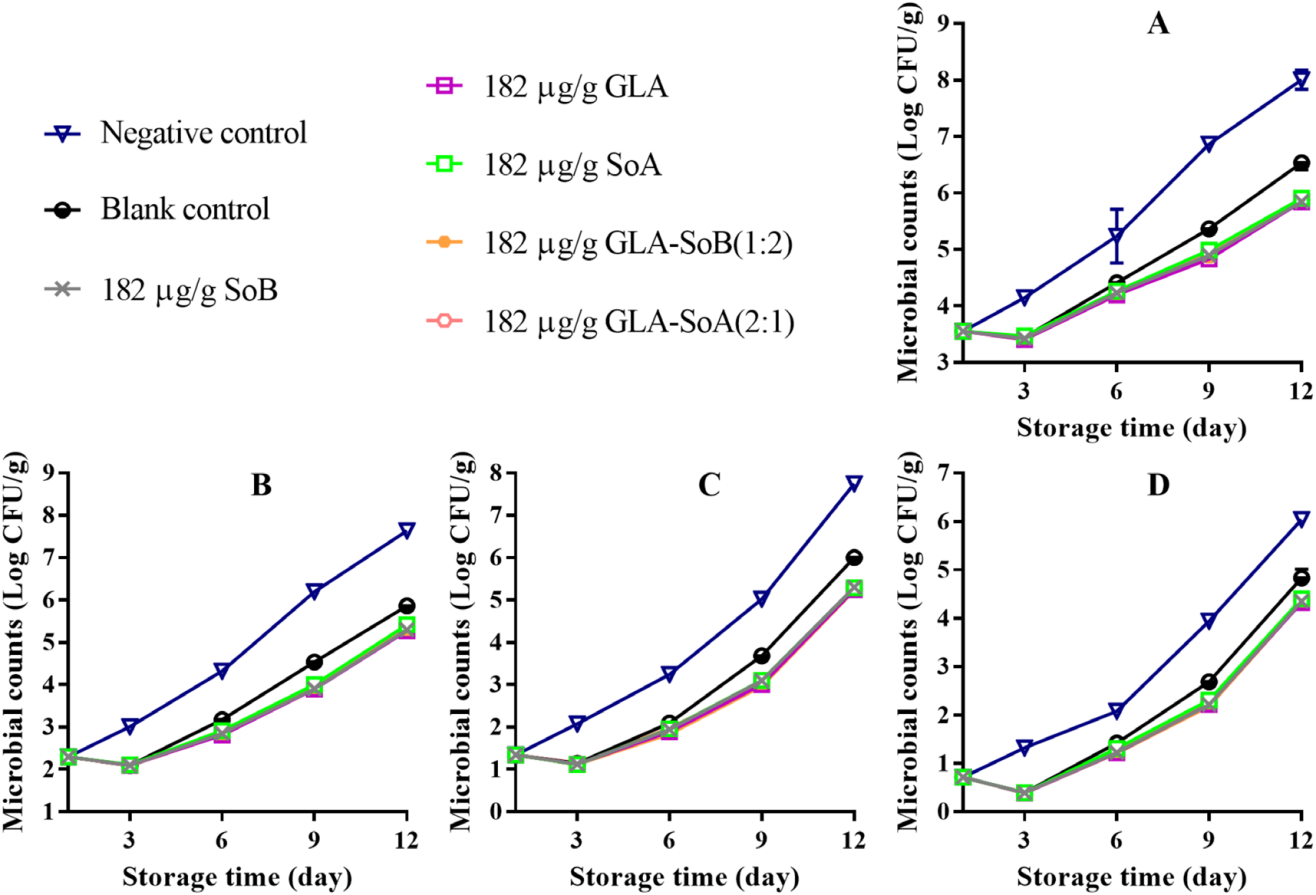
Assessment of total viable count (A), psychrophilic bacteria count (B), yeasts and molds count (C), and Enterobacteriaceae count (D) in minced salmon treated with glutaric acid (GLA), sodium bisulphite (SoB), sorbic acid (SoA) and their combinations at 182 μg/g during storage at 4°C. The blank control group represents minced salmon treated solely with 40% ethanol. Values represent the mean ± SD of three replicates.

Over a 12‐day storage period, microbial analysis revealed variations in TVCs, PTCs, Enterobacteriaceae counts, and yeast and mold counts among the minced salmon samples stored at 4°C (Figures 7, 8, 9, 10). Initially, the microbial counts for the blank control and experimental groups were reduced following the addition of 40% ethanol, tested substances, and their combinations. However, these counts increased progressively with storage time. The TVCs, PTCs, and yeast and mold counts of the negative control samples exceeded the acceptable limit around days 9 to 10. In contrast, blank control and experimental group samples maintained microbial counts below the acceptable limit throughout the 12‐day storage period. Notably, the experimental group samples exhibited significantly (*p* < 0.05) lower microbial counts compared to the blank control group. This reduction can be attributed to the inhibitory effects of the tested combinations, which likely extended the lag phase of microbial growth. Furthermore, the inhibitory effect became more pronounced with higher concentrations of the substances, effectively delaying microbial proliferation and preserving the quality of the salmon during refrigerated storage. Similar results were reported by Eghbalian et al. (2021), who demonstrated reduced microbial counts in trout treated with sodium caseinate‐gelatin combined with magnesium oxide (MgO) and spearmint ( *Mentha spicata* L.) essential oil. Likewise, Wu et al. (2016) periodically assessed the TVC and PTC in silver pomfret (*Pampus argentus*) samples treated with a chitosan‐gallic acid (CS‐GA) conjugate (chitosan gallate, CS‐g‐GA) during refrigerated storage at 4°C for 15 days. Their findings demonstrated that CS‐g‐GA exhibited higher antimicrobial activity, resulting in microbial populations 1.50–3.00 log_10_ CFU/g lower than those in control samples after 15 days of storage. These results are consistent with the findings of this study.

**FIGURE 11:**
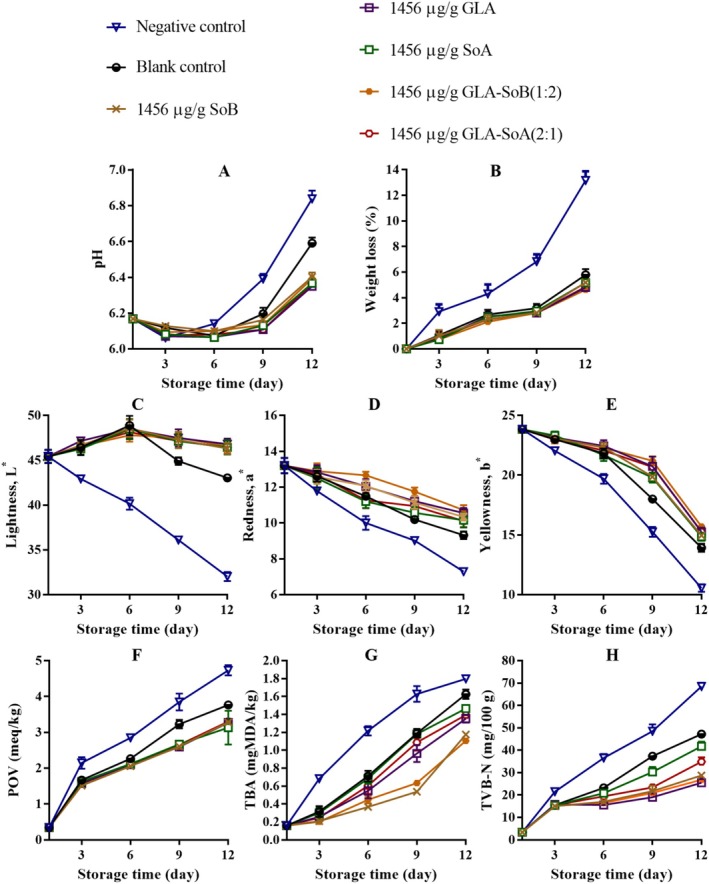
Assessment of pH (A), weight loss (B), lightness (C), redness (D), yellowness (E), peroxide value (POV) (F), thiobarbituric acid (TBA) value (G), and total volatile basic‐nitrogen (TVB‐N) (H) of minced salmon treated with glutaric acid (GLA), sodium bisulphite (SoB), sorbic acid (SoA) and their combinations at 1456 μg/g during storage at 4°C. The blank control group represents minced salmon treated solely with 40% ethanol. Values represent the mean ± SD of three replicates.

**FIGURE 12:**
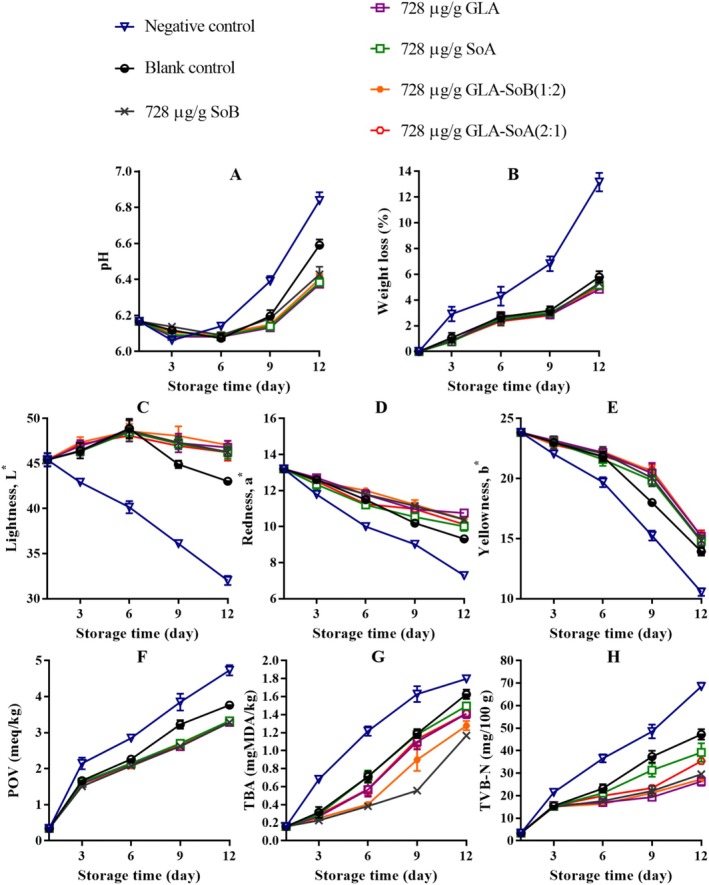
Assessment of pH (A), weight loss (B), lightness (C), redness (D), yellowness (E), peroxide value (POV) (F), thiobarbituric acid (TBA) value (G), and total volatile basic‐nitrogen (TVB‐N) (H) of minced salmon treated with glutaric acid (GLA), sodium bisulphite (SoB), sorbic acid (SoA) and their combinations at 728 μg/g during storage at 4°C. The blank control group represents minced salmon treated solely with 40% ethanol. Values represent the mean ± SD of three replicates.

**FIGURE 13:**
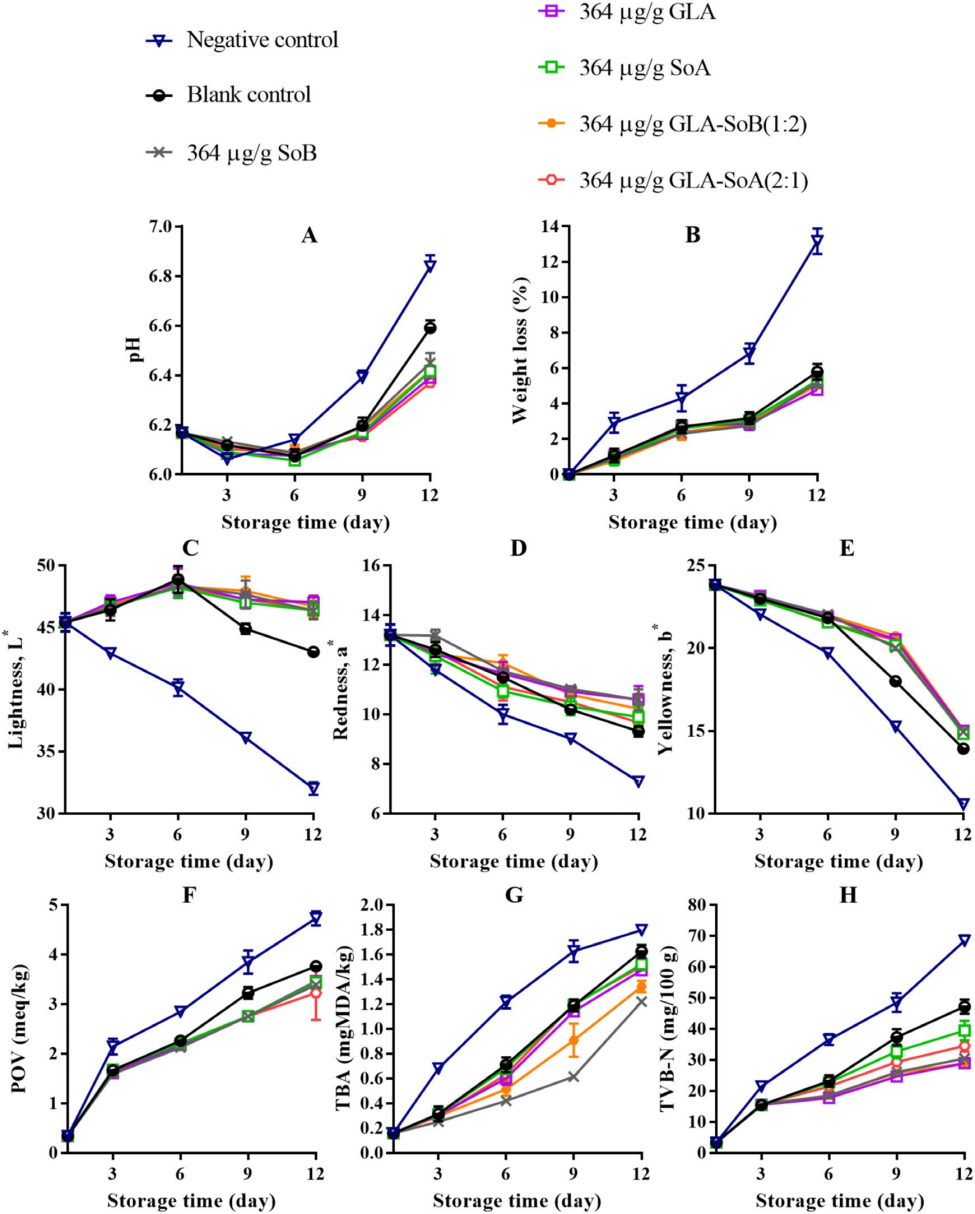
Assessment of pH (A), weight loss (B), lightness (C), redness (D), yellowness (E), peroxide value (POV) (F), thiobarbituric acid (TBA) value (G), and total volatile basic‐nitrogen (TVB‐N) (H) of minced salmon treated with glutaric acid (GLA), sodium bisulphite (SoB), sorbic acid (SoA) and their combinations at 364 μg/g during storage at 4°C. The blank control group represents minced salmon treated solely with 40% ethanol. Values represent the mean ± SD of three replicates.

**FIGURE 14:**
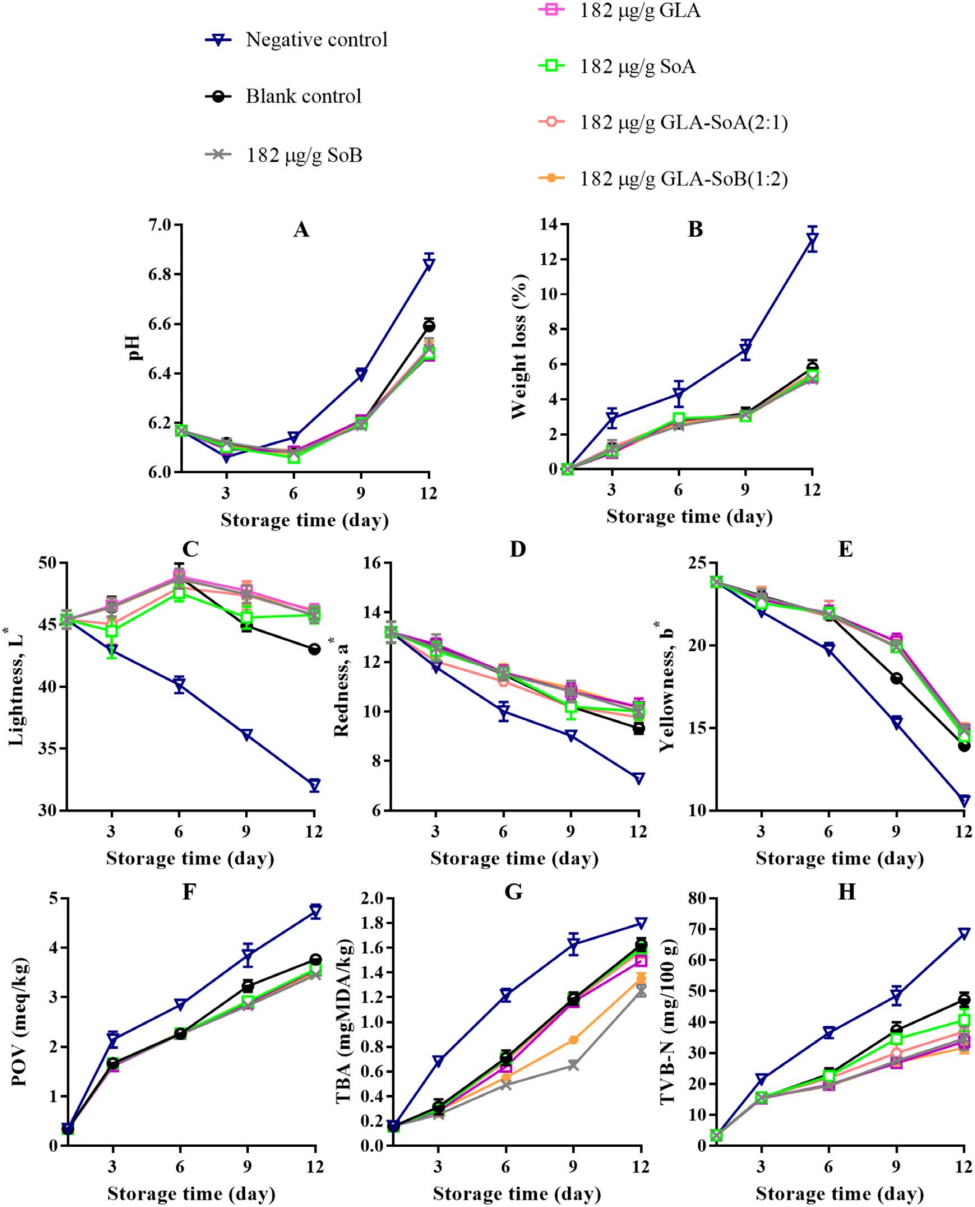
Assessment of pH (A), weight loss (B), lightness (C), redness (D), yellowness (E), peroxide value (POV) (F), thiobarbituric acid (TBA) value (G), and total volatile basic‐nitrogen (TVB‐N) (H) of minced salmon treated with glutaric acid (GLA), sodium bisulphite (SoB), sorbic acid (SoA) and their combinations at 182 μg/g during storage at 4°C. The blank control group represents minced salmon treated solely with 40% ethanol. Values represent the mean ± SD of three replicates.

The application of the tested combinations, along with 40% ethanol, significantly (*p* < 0.05) affected the pH of minced salmon meat during refrigerated storage (Figures 11A, 12A, 13A and 14A). The initial pH of the minced salmon was 6.17. The observed increase in pH during the storage period can be attributed to the production of basic compounds, such as ammonia, trimethylamine, and other biogenic amines, by spoilage bacteria (Jia et al. 2021). The combined antioxidant and antimicrobial properties of GLA‐SoB (1:2) effectively inhibited microbial growth, which resulted in a comparatively lower pH in the treated minced salmon. Similarly, GLA‐SoA (2:1), employing distinct antimicrobial mechanisms, also suppressed microbial proliferation, reducing the pH in treated minced salmon by the end of the 12‐day storage period. These findings highlight the efficacy of these combinations in mitigating microbial activity and maintaining product quality during refrigeration.

As depicted in Figures 11B, 12B, 13B and 14B, a reduction in weight loss was observed across all samples during storage. Notably, the negative control exhibited a significantly (*p* < 0.05) higher weight loss compared to the treated minced salmon. However, no significant (*p* > 0.05) differences in weight loss were observed among minced salmon treated with GLA, SoB, SoA, GLA‐SoA (2:1), and GLA‐SoB (1:2), though higher concentrations of these treatments demonstrated a more pronounced reduction in weight loss. The reduced weight loss in treated minced salmon can be attributed to the decreased myosin denaturation, which was effectively mitigated by the protective effects of the treatments (Wu et al. 2016). Myosin denaturation reduces the hydration capacity of muscle proteins, leading to increased water loss. Given that myofibrils constitute a substantial portion of muscle structure, alterations in water distribution within the tissue result in the loss of loosely bound water, contributing to greater weight loss in untreated samples (Wu et al. 2016). Furthermore, the lower microbiological degradation observed in treated minced salmon further reduced weight loss during refrigerated storage.

The impact of the tested combinations on the color parameters of minced salmon samples is depicted in Figures 11C−E, 12C−E, 13C−E, and 14C−E. The negative control group samples exhibited significantly lower (*p* < 0.05) L* values at the end of storage, likely attributable to the influence of sodium nitrate on the minced salmon. In contrast, the treated minced salmon demonstrated a significant (*p* < 0.05) preservation of L* values relative to the untreated minced salmon (negative control). Regarding a* value, the GLA‐SoA (2:1) and GLA‐SoB (1:2) treated minced salmon exhibited comparable values to those treated with the substances individually. This higher a* value can be attributed to the antioxidant or antimicrobial properties of the utilized substances. A significant (*p* < 0.05) elevation in a* value was observed in all treated samples compared to the blank control. For b* value, the lowest values (*p* < 0.05) were recorded in the negative control. The incorporation of dual combinations resulted in an increase in the b* values in the minced salmon, although no significant (*p* > 0.05) variation was observed among the experimental groups. These findings align with previous studies that reported comparable results in treated salmon (Fang et al. 2022; Lerfall et al. 2016).

The changes in the POV of minced salmon treated with dual combinations are presented in Figures 11F, 12F, 13F, and 14F. The untreated salmon samples exhibited the highest (*p* < 0.05) POV, indicating a more pronounced oxidative process. Conversely, minced salmon treated with the dual combinations showed significantly (*p* < 0.05) lower POVs compared to the blank control, demonstrating their efficacy in reducing lipid oxidation. The lipid oxidation, measured by TBA (Figures 11G, 12G, 13G, and 14G). Among the treatments, the SoB and GLA‐SoB (1:2) groups exhibited the lowest (*p* < 0.05) TBA values, indicating better protection against secondary lipid oxidation. In the negative control group, the oxidative reaction was significantly (*p* < 0.05) more pronounced, even compared to the blank control, underscoring the protective effects of the tested substances. TVB‐N's values increased progressively over time in all groups (Figures 11H, 12H, 13H, and 14H). Significant differences (*p* < 0.05) emerged between the control and treated groups after day 3 of storage. The increase in TVB‐N was markedly slower in minced salmon treated with the dual combinations. On days 6 and 9, the TVB‐N values of the negative and blank controls reached 35 mg/100 g (maximum threshold). By day 12, the treated groups exhibited significantly (*p* < 0.05) lower TVB‐N values than the two controls, attributable to their high antimicrobial activity and ability to retard microbial decomposition of proteins.

Minced salmon presents distinct storage challenges compared to sliced salmon, necessitating the use of more potent preservatives. The larger surface area of minced salmon exposes it to more oxygen molecules, accelerating lipid oxidation and spoilage (McMillin 2008). Furthermore, the grinding process distributes bacteria from the meat's surface throughout the product, significantly elevating the microbial load and spoilage rate. Minced salmon retains more moisture, promoting microbial growth and enzymatic activity (Nychas et al. 2008). Additionally, the physical breakdown of muscle fibers during grinding disrupts structural integrity, rendering proteins and lipids more susceptible to oxidative and enzymatic degradation (McMillin 2008). To address these challenges, preservatives for minced salmon must effectively inhibit higher microbial loads and target microbial distribution within the salmon meat matrix. Strong antioxidant properties are also crucial to mitigate lipid and protein oxidation caused by increased oxygen exposure. Dual combinations of antimicrobial and antioxidant substances, such as GLA and SoB, or enhanced antimicrobial systems like GLA and SoA, are highly effective choices. These dual combinations provide comprehensive protection against spoilage and oxidation, ensuring the safety and quality of minced salmon during storage.

## 4 Conclusions

Based on the results of MIC and ∑FIC, the tested combined GLA with SoA or SoB exhibited antimicrobial activity. GLA‐SoB combinations exhibited powerful antioxidant properties. The synergistic antimicrobial effects of these dual combinations were further validated through two model studies using sliced and minced salmon. In comparison to the negative control, treatments with GLA‐SoB (1:2) and GLA‐SoA (2:1) significantly (*p* < 0.05) reduced microbial growth, TVB‐N formation, lipid oxidation, and weight loss in both model studies during refrigerated storage. These treatments extended the shelf life of the salmon by at least 3 days. Furthermore, these treatments maintained higher color preservation and more stable pH levels over a more extended period than the control groups. At higher concentrations, the dual combinations exhibited enhanced antimicrobial activity. GLA offers a balanced preservation approach by combining cost‐effectiveness with efficacy, thereby reducing the reliance on SoB and SoA. The reduction in the use of these commercial preservatives mitigates potential risks associated with these preservatives, enhances safety, and positions the dual combinations as promising candidates for commercial applications in other meat products.

## Author Contributions

**Zhengrui Liao:** conceptualization (equal), validation (equal), visualization (equal), writing – original draft (equal), writing – review and editing (equal). **Xiaotong Zhu:** writing – review and editing (equal). **Thaigarajan Parumasivam:** data curation (equal), investigation (equal), methodology (equal). **Thuan‐Chew Tan:** writing – review and editing (equal). **Mohammad Alrosan:** writing – review and editing (equal). **Muhammad H. Alu' datt:** writing – review and editing (equal). **Ali Madi Almajwal:** writing – review and editing (equal).

## Conflicts of Interest

The authors declare no conflicts of interest.

## Data Availability

Data are available to the corresponding author upon reasonable request.

## References

[fsn370242-bib-0001] Abdel‐Wahab, M. , S. A. El‐Sohaimy , H. A. Ibrahim , and H. S. A. El‐Makarem . 2020. “Evaluation the Efficacy of Clove, Sage and Kiwifruit Peels Extracts as Natural Preservatives for Fish Fingers.” Annals of Agricultural Sciences 65: 98–106. 10.1016/j.aoas.2020.06.002.

[fsn370242-bib-0002] Alves, V. L. C. D. , B. P. M. Rico , R. M. S. Cruz , A. A. Vicente , I. Khmelinskii , and M. C. Vieira . 2018. “Preparation and Characterization of a Chitosan Film With Grape Seed Extract‐Carvacrol Microcapsules and Its Effect on the Shelf‐Life of Refrigerated Salmon (*Salmo salar*).” LWT‐ Food Science and Technology 89: 525–534. 10.1016/j.lwt.2017.11.013.

[fsn370242-bib-0003] Anas, M. , S. Ahmad , and A. Malik . 2019. “Microbial Escalation in Meat and Meat Products and Its Consequences.” In Health and Safety Aspects of Food Processing Technologies, 29–49. Springer. 10.1007/978-3-030-24903-8_3.

[fsn370242-bib-0004] Basavegowda, N. , and K. H. Baek . 2021. “Synergistic Antioxidant and Antibacterial Advantages of Essential Oils for Food Packaging Applications.” Biomolecules 11: 1267. 10.3390/biom11091267. 34572479 PMC8466708

[fsn370242-bib-0005] Ben Akacha, B. , J. Švarc‐Gajić , K. Elhadef , et al. 2022. “The Essential Oil of Tunisian Halophyte *Lobularia maritima* : A Natural Food Preservative Agent of Ground Beef Meat.” Lifestyles 12: 1571. 10.3390/life12101571. PMC9605339 36295006

[fsn370242-bib-0006] Ben Braïek, O. , and S. Smaoui . 2021. “Chemistry, Safety, and Challenges of the Use of Organic Acids and Their Derivative Salts in Meat Preservation.” Journal of Food Quality 2021: 6653190. 10.1155/2021/6653190.

[fsn370242-bib-0007] Brennan, M. , G. Le Port , A. Pulvirenti , and R. Gormley . 1999. “The Effect of Sodium Metabisulphite on the Whiteness and Keeping Quality of Sliced Mushrooms.” LWT‐ Food Science and Technology 32: 460–463. 10.1006/fstl.1999.0575.

[fsn370242-bib-0008] Chaichi, M. , A. Mohammadi , F. Badii , and M. Hashemi . 2021. “Triple Synergistic Essential Oils Prevent Pathogenic and Spoilage Bacteria Growth in the Refrigerated Chicken Breast Meat.” Biocatalysis and Agricultural Biotechnology 32: 101926. 10.1016/j.bcab.2021.101926.

[fsn370242-bib-0009] Chen, J. , Y. Zheng , Q. Kong , Z. Sun , and X. Liu . 2023. “A Wechat Miniprogram (‘Fresh Color’) Based on Smart Phone to Indicate the Freshness of Atlantic Salmon (* Salmo salar L*.) and Oysters on Site by Detection of the Color Changes of Curcumin Films.” Food Control 145: 109520. 10.1016/j.foodcont.2022.109520.

[fsn370242-bib-0010] Choi, J. H. , D. H. Song , J. S. Hong , et al. 2019. “Nitrite Scavenging Impact of Fermented Soy Sauce *In Vitro* and in a Pork Sausage Model.” Meat Science 151: 36–42. 10.1016/j.meatsci.2019.01.001. 30685509

[fsn370242-bib-0011] Christensen, L. B. , M. B. Hovda , and T. M. Rode . 2017. “Quality Changes in High Pressure Processed Cod, Salmon and Mackerel During Storage.” Food Control 72: 90–96. 10.1016/j.foodcont.2016.07.037.

[fsn370242-bib-0012] Comi, G. 2017. “Spoilage of Meat and Fish.” In The Microbiological Quality of Food, edited by A. Bevilacqua , M. R. Corbo , and M. Sinigaglia , 179–210. Woodhead Publishing Ltd.

[fsn370242-bib-0013] Dhakal, J. , C. G. Aldrich , and C. Knueven . 2019. “Assessing the Efficacy of Sodium Bisulfate and Organic Acid Treatments for Control of *Salmonella typhimurium* in Rendered Chicken Fat Applied to Pet Foods.” Journal of Food Protection 82: 1864–1869. 10.4315/0362-028X.JFP-18-560. 31613163

[fsn370242-bib-0014] Dirpan, A. , and S. H. Hidayat . 2023. “Quality and Shelf‐Life Evaluation of Fresh Beef Stored in Smart Packaging.” Food 12: 396. 10.3390/foods12020396. PMC9857838 36673488

[fsn370242-bib-0015] Dittoe, D. K. , J. A. Atchley , K. M. Feye , J. A. Lee , C. J. Knueven , and S. C. Ricke . 2019. “The Efficacy of Sodium Bisulfate Salt (SBS) Alone and Combined With Peracetic Acid (PAA) as an Antimicrobial on Whole Chicken Drumsticks Artificially Inoculated With *Salmonella enteritidis* .” Frontiers in Veterinary Science 6: 6. 10.3389/fvets.2019.00006. 30761312 PMC6363672

[fsn370242-bib-0016] Domínguez, R. , M. Pateiro , P. E. Munekata , et al. 2021. “Protein Oxidation in Muscle Foods: A Comprehensive Review.” Antioxidants (Basel) 11, no. 60: 60. 10.3390/antiox11010060. 35052564 PMC8773412

[fsn370242-bib-0017] Eghbalian, M. , N. Shavisi , Y. Shahbazi , and F. Dabirian . 2021. “Active Packaging Based on Sodium Caseinate‐Gelatin Nanofiber Mats Encapsulated With *Mentha spicata* L. Essential Oil and MgO Nanoparticles: Preparation, Properties, and Food Application.” Food Packaging and Shelf Life 29: 100737. 10.1016/j.fpsl.2021.100737.

[fsn370242-bib-0018] Fang, M. , R. Wang , A. K. Agyekumwaa , Y. Yu , and X. Xiao . 2022. “Antibacterial Effect of Phenyllactic Acid Against *Vibrio parahaemolyticus* and Its Application on Raw Salmon Fillets.” LWT 154: 112586. 10.1016/j.lwt.2021.112586.

[fsn370242-bib-0019] Hartwig, A. , M. Arand , and Commission, M . 2020. Glutaric acid. The MAK Collection for Occupational Health and Safety, 5, Doc053.

[fsn370242-bib-0020] Hlima, H. B. , S. Smaoui , M. Barkallah , et al. 2021. “Sulfated Exopolysaccharides From *Porphyridium Cruentum*: A Useful Strategy to Extend the Shelf Life of Minced Beef Meat.” International Journal of Biological Macromolecules 193: 1215–1225. 10.1016/j.ijbiomac.2021.10.161. 34717983

[fsn370242-bib-0021] Hsouna, A. B. , A. Boye , B. B. Ackacha , et al. 2022. “Thiamine Demonstrates Bio‐Preservative and Anti‐Microbial Effects in Minced Beef Meat Storage and Lipopolysaccharide (LPS)‐Stimulated RAW 264.7 Macrophages.” Animals 12: 1646. 10.3390/ani12131646. 35804544 PMC9264808

[fsn370242-bib-0022] İrkin, R. , N. Degirmencioglu , and M. Guldas . 2015. “Effects of Organic Acids to Prolong the Shelf‐Life and Improve the Microbial Quality of Fresh Cut Broccoli Florets.” Quality Assurance & Safety of Crops and Food 7: 737–745. 10.3920/QAS2014.0489.

[fsn370242-bib-0023] Ismail, I. , and N. Huda . 2024. “Current Techniques and Technologies of Meat Quality Evaluation.” In Hand Book of Processed Functional Meat Products, edited by S. A. Rather and F. A. Masoodi , 437–512. Springer.

[fsn370242-bib-0024] Jia, Z. , C. Shi , J. Zhang , and Z. Ji . 2021. “Comparison of Freshness Prediction Method for Salmon Fillet During Different Storage Temperatures.” Journal of the Science of Food and Agriculture 101: 4987–4994. 10.1002/jsfa.11142. 33543483

[fsn370242-bib-0025] Karami, N. , A. Kamkar , Y. Shahbazi , and A. Misaghi . 2020. “Effects of Active Chitosan‐Flaxseed Mucilage‐Based Films on the Preservation of Minced Trout Fillets: A Comparison Among Aerobic, Vacuum, and Modified Atmosphere Packaging.” Packaging Technology and Science 33: 469–484. 10.1002/pts.2530.

[fsn370242-bib-0026] Kim, S. , and M. Rhee . 2013. “Marked Synergistic Bactericidal Effects and Mode of Action of Medium‐Chain Fatty Acids in Combination With Organic Acids Against *Escherichia Coli* O157: H7.” Applied and Environmental Microbiology 79: 6552–6560. 10.1128/AEM.02164-13. 23956396 PMC3811494

[fsn370242-bib-0027] Krishnamoorthy, R. , M. A. Gassem , J. Athinarayanan , V. S. Periyasamy , S. Prasad , and A. A. Alshatwi . 2021. “Antifungal Activity of Nanoemulsion From *Cleome viscosa* Essential Oil Against Food‐Borne Pathogenic *Candida albicans* .” Saudi Journal of Biological Sciences 28: 286–293. 10.1016/j.sjbs.2020.10.001. 33424308 PMC7785440

[fsn370242-bib-0028] Lamas, A. , J. M. Miranda , B. Vázquez , A. Cepeda , and C. M. Franco . 2016. “An Evaluation of Alternatives to Nitrites and Sulfites to Inhibit the Growth of *Salmonella Enterica* and *Listeria Monocytogenes* in Meat Products.” Food 5: 74. 10.3390/foods5040074. PMC5302421 28231169

[fsn370242-bib-0029] Lerfall, J. , E. Å. Bendiksen , J. V. Olsen , and M. Østerlie . 2016. “A Comparative Study of Organic‐Versus Conventional Atlantic Salmon. II. Fillet Color, Carotenoid‐and Fatty Acid Composition as Affected by Dry Salting, Cold Smoking and Storage.” Aquaculture 451: 369–376. 10.1016/j.aquaculture.2015.10.004.

[fsn370242-bib-0030] Li, B. , X. Wang , X. Gao , et al. 2021. “Shelf‐Life Extension of Large Yellow Croaker (*Larimichthys crocea*) Using Active Coatings Containing Lemon Verbena (*Lippa citriodora* Kunth.) Essential Oil.” Frontiers in Nutrition 8: 678643. 10.3389/fnut.2021.678643. 34355009 PMC8329554

[fsn370242-bib-0031] Liao, Z. , Y. K. Yeoh , T. Parumasivam , et al. 2024. “Medium‐Chain Dicarboxylic Acids: Chemistry, Pharmacological Properties, and Applications in Modern Pharmaceutical and Cosmetics Industries.” RSC Advances 14: 17008–17021. 10.1039/D4RA02598A. 38808239 PMC11130641

[fsn370242-bib-0032] Manihuruk, F. M. , T. Suryati , and I. I. Arief . 2017. “Effectiveness of the Red Dragon Fruit ( *Hylocereus polyrhizus* ) Peel Extract as the Colorant, Antioxidant, and Antimicrobial on Beef Sausage.” Media Peternakan 40: 47–54.

[fsn370242-bib-0033] Mavani, N. R. , J. M. Ali , M. Hussain , N. A. Rahman , and H. Hashim . 2024. “Determining Food Safety in Canned Food Using Fuzzy Logic Based on Sulphur Dioxide, Benzoic Acid and Sorbic Acid Concentration.” Heliyon 10: e26273. 38384537 10.1016/j.heliyon.2024.e26273 PMC10879013

[fsn370242-bib-0034] McMillin, K. W. 2008. “Where Is MAP Going? A Review and Future Potential of Modified Atmosphere Packaging for Meat.” Meat Science 80: 43–65. 10.1016/j.meatsci.2008.05.028. 22063169

[fsn370242-bib-0035] Meyer, A. , K. Suhr , P. Nielsen , and F. Holm . 2002. “Minimal Processing Technologies in the Food Industries.” In Natural Food Preservatives, vol. 8, 124–174. Woodhead Publishing.

[fsn370242-bib-0036] Molina, B. , M. Sáez , T. Martínez , J. Guil‐Guerrero , and M. Suárez . 2014. “Effect of Ultraviolet Light Treatment on Microbial Contamination, Some Textural and Organoleptic Parameters of Cultured Sea Bass Fillets (*Dicentrarchus Labrax*).” Innovative Food Science & Emerging Technologies 26: 205–213. 10.1016/j.ifset.2014.07.002.

[fsn370242-bib-0037] Nagasinduja, V. , and S. Shahitha . 2019. “Food Preservatives‐Types, Uses and Side Effects.” International Journal of Basic and Applied Research 9: 632–637.

[fsn370242-bib-0038] National Health Commission of the People's Republic of China , and State Administration for Market Regulation . 2024. National Food Safety Standard for the Use of Food Additives. China State Administration of Market Supervision and Regulation.

[fsn370242-bib-0039] Nychas, G. J. E. , P. N. Skandamis , C. C. Tassou , and K. P. Koutsoumanis . 2008. “Meat Spoilage During Distribution.” Meat Science 78: 77–89. 10.1016/j.meatsci.2007.06.020. 22062098

[fsn370242-bib-0040] Olivas, G. I. , J. J. Rodriguez , and G. V. Barbosa‐Canovas . 2003. “Edible Coatings Composed of Methylcellulose, Stearic Acid, and Additives to Preserve Quality of Pear Wedges.” Journal of Food Processing and Preservation 27: 299–320. 10.1111/j.1745-4549.2003.tb00519.x.

[fsn370242-bib-0041] Pakawatchai, C. , S. Siripongvutikorn , and W. Usawakesmanee . 2009. “Effect of Herb and Spice Pastes on the Quality Changes in Minced Salmon Flesh Waste During Chilled Storage.” Asian Journal of Food and Agro‐Industry 2: 481–492.

[fsn370242-bib-0042] Pardeshi, K. A. , S. R. Malwal , A. Banerjee , S. Lahiri , R. Rangarajan , and H. Chakrapani . 2015. “Thiol Activated Prodrugs of Sulfur Dioxide (SO_2_) as MRSA Inhibitors.” Bioorganic & Medicinal Chemistry Letters 25: 2694–2697. 10.1016/j.bmcl.2015.04.046. 25981687

[fsn370242-bib-0043] Quoc, L. P. T. 2018. “Antimicrobial Activity of Preservatives in Food Technology.” Agricultural Food Engineering 11: 164–170.

[fsn370242-bib-0044] Rojo‐Bezares, B. , Y. Sáenz , M. Zarazaga , C. Torres , and F. Ruiz‐Larrea . 2007. “Antimicrobial Activity of Nisin Against *Oenococcus oeni* and Other Wine Bacteria.” International Journal of Food Microbiology 116: 32–36. 10.1016/j.ijfoodmicro.2006.12.020. 17320991

[fsn370242-bib-0045] Seo, Y. , M. Sung , J. Hwang , and Y. Yoon . 2023. “Minimum Inhibitory Concentration (MIC) of Propionic Acid, Sorbic Acid, and Benzoic Acid Against Food Spoilage Microorganisms in Animal Products to Use MIC as Threshold for Natural Preservative Production.” Food Science of Animal Resources 43: 319–330. 10.5851/kosfa.2022.e79. 36909850 PMC9998193

[fsn370242-bib-0046] Stopforth, J. , and T. Kudron . 2020. “Sorbic Acid and Sorbates.” In Antimicrobials in Foods, edited by P. M. Davidson , J. N. Sofos , and A. L. Branen , 49–59. CRC Press.

[fsn370242-bib-0047] Vellanki, B. P. , B. Batchelor , and A. Abdel‐Wahab . 2013. “Advanced Reduction Processes: A New Class of Treatment Processes.” Environmental Engineering Science 30: 264–271. 10.1089/ees.2012.0273. 23840160 PMC3696927

[fsn370242-bib-0048] Wu, C. , S. Fu , Y. Xiang , et al. 2016. “Effect of Chitosan Gallate Coating on the Quality Maintenance of Refrigerated (4°C) Silver Pomfret (*Pampus argentus*).” Food and Bioprocess Technology 9: 1835–1843. 10.1007/s11947-016-1771-5.

[fsn370242-bib-0049] Xin, L. Y. , T. H. Min , P. N. L. M. Zin , T. Pulingam , J. N. Appaturi , and T. Parumasivam . 2021. “Antibacterial Potential of Malaysian Ethnomedicinal Plants Against Methicillin‐Susceptible *Staphylococcus Aureus* (MSSA) and Methicillin‐Resistant *Staphylococcus Aureus* (MRSA).” Saudi Journal of Biological Sciences 28: 5884–5889. 10.1016/j.sjbs.2021.06.036. 34588904 PMC8459151

[fsn370242-bib-0050] Yan, Q. , L. Wang , X. Sun , et al. 2022. “Improvement in the Storage Quality of Fresh Salmon ( *Salmo salar* ) Using a Powerful Composite Film of Rice Protein Hydrolysates and Chitosan.” Food Control 142: 109211. 10.1016/j.foodcont.2022.109211.

